# Dissolved organic matter-generated photoelectrons enable microbial antimonate reduction in mine stream sediments

**DOI:** 10.1038/s41467-026-72108-1

**Published:** 2026-04-20

**Authors:** Linao Zhu, Hanbing Gao, Min Shen, Kuanxin Huang, Yongqun Tang, Lele He, Jing Huang, Cheng Zhao, Weiping Xiong, Xiaoli Ni, Honghui Wu, Shikai Li, Zhaohui Guo, Jie Cao, Wenjing Xue, Rui Xu

**Affiliations:** 1 Institute of Environmental Engineering, School of Metallurgy and Environment, Central South University, Changsha, PR China; 2 Urban Geological Survey and Monitor Institute of Hunan Province, Changsha, PR China; 3 College of Environmental Science and Engineering, Hunan University, Changsha, PR China; 4 State Key Laboratory of Woody Oil Resources Utilization, Hunan Academy of Forestry, Changsha, PR China; 5 College of Environmental Science and Engineering, Yangzhou University, Yangzhou, PR China

**Keywords:** Environmental impact, Element cycles

## Abstract

Antimony contamination in mining-impacted river basins poses persistent environmental risks, yet the microbial processes governing antimony redox transformation under anoxic sediments remain poorly understood. Here, we report that photoelectrons generated by naturally occurring dissolved organic matter can support microbial antimonate reduction. Natural dissolved organic matter exhibits sustained photocurrent responses under illumination. In anoxic microcosms, indigenous sediment communities achieve 50–70% antimonate reduction with photoelectron supply, thereby constraining purely abiotic reduction or alternative electron-donor explanations under our experimental conditions. Multi-omics further identify non-phototrophic taxa (e.g., *Sphingomonas* and *Bosea*) with elevated antimony reduction/detoxification pathways and respiratory electron-transfer components under photoelectron exposure. The consistent presence of photosensitive dissolved organic matter and candidate taxa across mining-impacted sediments suggests broader environmental relevance. These findings indicate that photosensitive dissolved organic matter may provide a photoelectron flux that influences microbial antimony redox transformations in anoxic sediments.

## Abstract (web summary)

Sunlight-exposed dissolved organic matter generates photoelectrons that microbes capture to reduce antimonate in anoxic sediments, revealing an overlooked electron supply route that may shape antimony cycling in mining-impacted streams.

## Introduction

China is among the world’s major producers of antimony (Sb)^1^. Large Sb deposits are concentrated in South China, where hilly–mountainous terrain and dense river networks promote downstream transport^2^. Weathering of sulfide ores, tailings leachates, and other mining activities release Sb that is readily mobilized by rainfall and runoff. As a result, Sb contamination has become a persistent environmental concern across mining-impacted river basins.

Microbial redox transformations strongly influence Sb mobility in aquatic systems^3,4^. Sb occurs mainly as pentavalent antimonate [Sb(V)] in oxic waters, whereas trivalent antimonite [Sb(III)] prevails in anoxic or suboxic sediments. Because Sb(III) can bind to sulfide minerals, iron phases and organic ligands by forming relatively low-mobility precipitates, thus microbial Sb(V) reduction may contribute to natural attenuation^5^. Despite its importance, the mechanisms of microbial Sb(V) reduction remain far less understood than those of the analogous arsenic (As) system^6^. To date, only a limited number of heterotrophic Sb(V)-reducing strains have been isolated. They use organic electron donors (e.g., lactate, acetate or citrate) to drive Sb(V) reduction^7–10^. A smaller number of autotrophic Sb(V) reducers that oxidize hydrogen or reduced sulfur compounds have also been reported^11–13^. Together, these microorganisms employ As-related enzymes such as the cytoplasmic reductases ArsC^14^, the respiratory ArrA^15^, or the recently described Sb-specific reductases AnrA^10^ and SbrA^16^. Consequently, heterotrophic metabolism in organic-rich sediments has often been considered as a major contributor to Sb(V) reduction^7^.

Dissolved organic matter (DOM) is one of Earth’s largest organic reservoirs and is ubiquitous in natural waters, sediments and soils^17^. DOM comprises a complex mixture of proteins, humic substances, carbohydrates, and small molecules with amide, carboxyl, hydroxyl, and quinone groups^18^. In sediments, DOM not only fuels heterotrophic microbes as a carbon and electron source^19^ but also mediates metal transformations through complexation, radical generation and electron shuttling^20^. Recent studies further show that DOM can act as a natural photosensitizer under sunlight exposure^18,21^. Photoexcited DOM produces reactive intermediates including photogenerated electrons^18^, and its electron-donating capacity correlates with redox-active structures^21^. Quinone- and phenolic-rich fractions often yield higher photoelectron fluxes under illumination^17^, highlighting DOM’s intrinsic photoelectrochemical activity and its potential influence on metal redox cycling.

These observations raise a central question: can DOM-derived photoelectrons drive microbial Sb(V) reduction? Early work in engineered biohybrid systems has shown that non-phototrophic bacteria can harvest photoelectrons from (semi)conductor materials to reduce CO^22^, CO_2_^23^, and nitrate^24^. This form of microbial photoelectrophy utilizes extracellular photoelectrons rather than photons (phototrophy) or organic substrates (chemotrophy)^25^. However, natural analogues in sediments remain largely undocumented^23,26^, and direct evidence for photoelectron-assisted Sb(V) reduction by indigenous microbes is limited.

Here, we report that anoxic, DOM-rich sediments exposed to sunlight can enable photoelectron-assisted microbial Sb(V) reduction (PESbR). This suggests PESbR may represent a low-input and self-sustaining pathway for Sb(V) reduction that can complement abiotic photochemical and engineered electrochemical pathways in natural systems. In this study, we aim to (1) characterize the photoelectrochemical responses of diverse DOM; (2) evaluate the ability of indigenous sediment communities to use photoelectrons for Sb(V) reduction; (3) identify non-phototrophic taxa that express Sb(V) reductases potentially involved in this process; and (4) survey the distribution of photosensitive DOM signatures and PESbR-associated microorganisms in mining-impacted sites (workflow in Supplementary Fig. 1). Given the limited diversity of known Sb(V) reducers and the complexity of Sb redox cycling, this study introduces PESbR as a form of microbial photoelectrophy and explores how DOM-driven photoelectrochemistry can affect redox dynamics and contaminant behavior in anoxic sediments.

## Results

### Photoelectrochemical activity of DOM

**Fig. 1:**
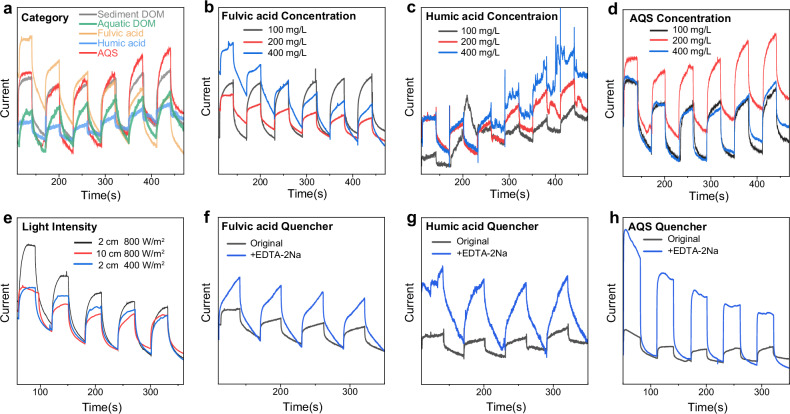
Photoelectrochemical activity of DOM under light–dark cycling. **a** Photocurrent responses of humic acid, fulvic acid, riverine aquatic DOM, sediment-derived DOM and the AQS (a proxy for DOM quinone-like moieties). All tested at 200 mg L⁻¹ and 800 W m⁻². **b**–**d** Effects of concentration (100–400 mg L⁻¹) on photocurrent generation. **e** Effects of irradiance (400–800 W m⁻²) and lamp distance (2–10 cm) on photocurrent generation. **f**–**h** Enhancement of photocurrents after adding the hole quencher.

To assess the prevalence of photosensitive DOM in sediments, we tested humic acid, fulvic acid, riverine DOM, sediment-derived DOM, together with a redox-active quinone model compound AQS, for photocurrent generation under simulated sunlight (800 W m⁻²). All tested samples produced immediate and reproducible photocurrent responses during light–dark cycling (Fig. 1a). AQS, sediment-derived DOM and fulvic acid yielded higher currents than riverine DOM and humic acid, suggesting that different DOM structures can act as photosensitizers with varying efficiencies. We then examined the effects of concentration (100–400 mg L⁻¹), irradiance (400–800 W m⁻²) and lamp distance (2–10 cm) on photocurrent generation (Fig. 1b–e). Photocurrent increased with irradiance and reached a maximum at 800 W m⁻² and 2 cm; whereas increasing concentration did not produce a proportional increase in photocurrent. Addition of the hole quencher EDTA-2Na further enhanced photocurrents across all tested samples (Fig. 1f–h), consistent with increased net photoelectron yield under hole scavenging conditions. Overall, these assays indicate that diverse DOM types possess intrinsic photoelectrochemical activity but depends on molecular composition, concentration and irradiation conditions.

### Photoelectrons enable microbial Sb(V) reduction

**Fig. 2:**
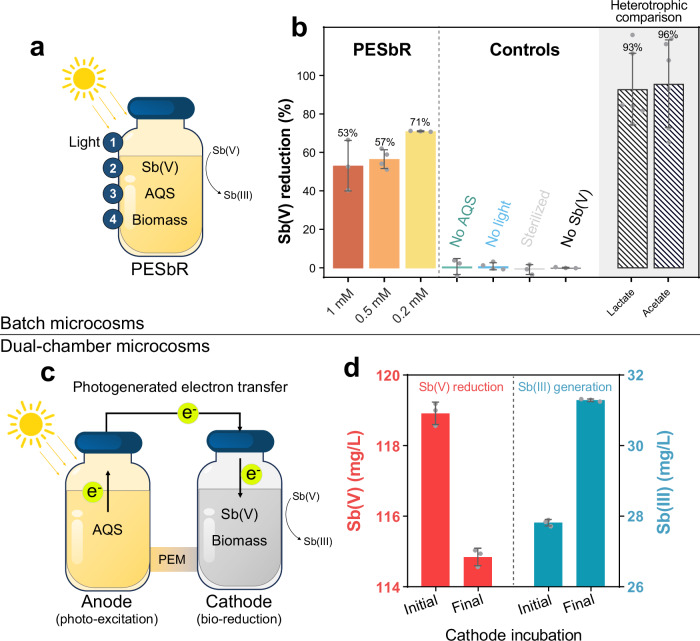
Photoelectron-assisted microbial Sb(V) reduction (PESbR). **a** Schematic of the batch microcosm where light, Sb(V), AQS (a proxy for quinone-like moieties in photosensitive DOM) and live biomass are all required to support PESbR. **b** Sb(V) reduction rates in the complete PESbR treatment compared with controls lacking one component (no AQS, no light, no added Sb(V) or sterile sediment) and with heterotrophic systems amended with lactate or acetate. **c** Schematic of the dual-chamber microcosm with an illuminated anode and a dark, anaerobic cathode containing Sb(V) and sediment slurry to test photoelectron transfer. **d** Photoelectrons generated from the illuminated anode drove Sb(V) reduction and Sb(III) generation in the cathode chamber. Data are presented as mean values ± SD.

We next tested whether indigenous sediment microorganisms can use illumination-generated photoelectrons to reduce Sb(V). Batch microcosms were constructed to include four key components: light, Sb(V), photosensitizer (AQS, used here as a quinone proxy for redox-active moieties in DOM), and active microbial biomass (Fig. 2a). Only the complete PESbR treatment showed rapid Sb(V) reduction (Fig. 2b). Over 7 days, reduction efficiencies reached 53%–71% at initial Sb(V) concentrations of 0.2–1 mM, corresponding to average volumetric rates of ~24–72 µmol Sb L⁻¹ d⁻¹. Sb(V) reduction was accompanied by Sb(III) accumulation, and dissolved total Sb remained nearly constant over time, suggesting limited sorption losses in microcosms (Supplementary Fig. 2). In contrast, removing any single factor resulted in negligible Sb(V) reduction ( < 1%). Meanwhile, microcosms amended with lactate/acetate showed highly efficient Sb(V) reduction (Fig. 2b), consistent with reported rates for classical heterotrophic reducers.

To decouple photoexcitation from direct microbial contact, we constructed a dual-chamber cell in which the anode chamber contained illuminated AQS, while the dark biocathode contained Sb(V) and sediment slurry under anaerobic conditions (Fig. 2c). Over nine days, photoelectron transfer led to a ~ 0.034 mM decrease in Sb(V) in biocathode, accompanied by a ~ 0.030 mM increase in Sb(III) (Fig. 2d). Although absolute reduction rates were lower than those in well-mixed batch microcosms, likely due to charge-transfer limitations across the proton-exchange membrane. Nevertheless, these results support that photogenerated electrons can be transferred to microbes and contribute to Sb(V) reduction. This finding also suggests the biological nature of PESbR rather than purely abiotic photoelectrochemical reduction^27^.

### Microbial community shifts under PESbR conditions

**Fig. 3:**
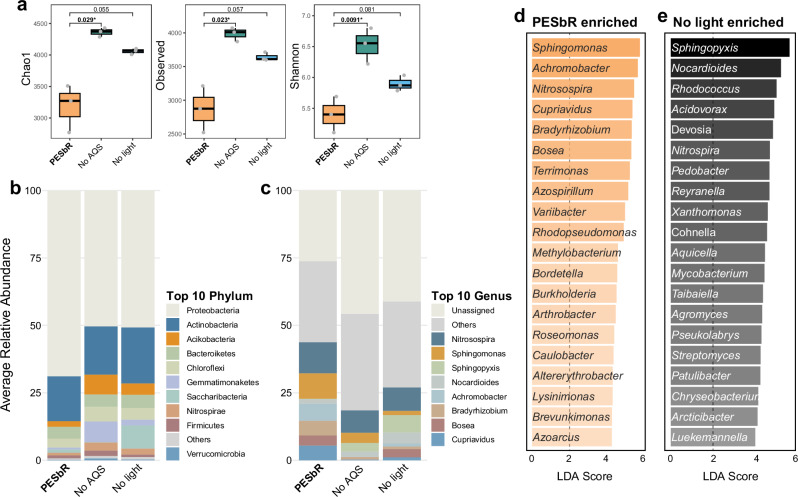
Microbial community responses in microcosms. **a** Alpha diversity indices (Chao1, Observed ASVs, Shannon) in complete PESbR microcosms versus controls. **b**, **c** Relative abundance of the ten most abundant phyla **b** and genera **c** across treatments. **d**, **e** LEfSe analysis (LDA > 2) showing the genera enriched (top 20) in PESbR microcosms **d** and those enriched in the dark controls representing putative conventional Sb(V) reducers **e**.

PESbR conditions imposed strong selection on the sediment microbiome. After 30 days, α-diversity indices (Chao1, observed species, Shannon) were significantly lower in PESbR microcosms (Fig. 3a), and β-diversity analyses showed clear separation among treatments (Supplementary Fig. 3). PESbR microcosms were dominated by *Proteobacteria* (68%) and *Actinobacteria* (17%) (Fig. 3b). Within these phyla, *Sphingomonas* (9%), *Achromobacter* (6%), *Cupriavidus* (5%), *Bradyrhizobium* (5%) and *Bosea* (4%) were specifically enriched (Fig. 3c). In contrast, the light-only controls were favored typical phototroph-associated groups such as *Cyanobacteria* and lineages *Gemmatimonadetes* (Supplementary Fig. 4). LEfSe analysis (LDA > 2; Fig. 3d, e) further confirmed these patterns by identifying PESbR-associated taxa that were absent or rare in all controls. Dark microcosms were dominated by genera commonly linked to Sb-tolerant and/or heterotrophic Sb(V) reduction, including *Sphingopyxis*, *Nocardioides*, *Rhodococcus*, *Acidovorax*, *Mycobacterium*, *Chryseobacterium*, and *Devosia*. In comparison, PESbR microcosms were enriched in *Sphingomonas*, *Achromobacter*, *Nitrosospira*, *Cupriavidus*, *Bradyrhizobium*, *Bosea*, *Azospirillum*, and *Methylobacterium*, several of which ranked among the top ten most abundant genera in PESbR microcosms (Fig. 3c). A comprehensive comparison of PESbR-enriched and dark-enriched genera is provided in Supplementary Data 1. The summed relative abundance of PESbR-associated genera reached ~60% in PESbR microcosms, but only ~20% in the AQS-free or light-free controls (Supplementary Fig. 5).

### Metagenomic reconstruction of PESbR-associated taxa

**Fig. 4:**
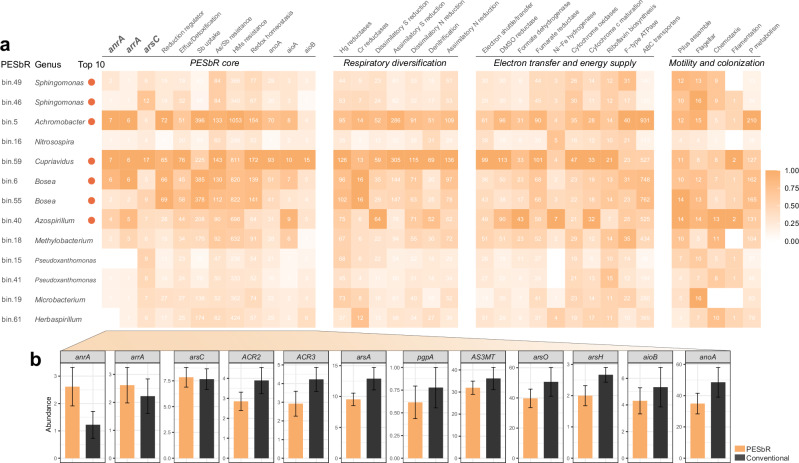
Metagenomic reconstruction of PESbR-associated taxa. **a** Heatmap showing the presence of core Sb(V)-reduction genes (e.g., *anrA*, *arrA*, *arsC*) and supporting PESbR modules across recovered MAGs. Taxa ranking among the top 10 in relative abundance (16S profiles) are highlighted with red circles. **b** Comparison of the prevalence of core Sb(V)-reduction features between PESbR MAGs (*n* = 13) and conventional Sb(V)-reducer MAGs (*n* = 9).

To link these community shifts to genetic potential, metagenomic binning recovered 13 MAGs (average completeness ~77%, contamination ~2%), representing 10 PESbR-enriched genera (Supplementary Data 2): *Sphingomonas* (bin.49, bin.46), *Cupriavidus* (bin.59), *Bosea* (bin.6, bin.55), *Azospirillum* (bin.40), *Achromobacter* (bin.5), *Pseudoxanthomonas* (bin.41, bin.15), *Nitrosospira* (bin.16), *Microbacterium* (bin.19), *Methylobacterium* (bin.18), and *Herbaspirillum* (bin.61). These MAGs correspond well with PESbR-associated taxa identified by LEfSe (Fig. 3d), and contained abundant PESbR-related genes (Fig. 4a, details in Supplementary Data 3).

#### Core Sb(V) reduction

All MAGs carried key genes associated with Sb(V) reduction and/or detoxification. The Sb(V)-specific respiratory reductase *anrA*, the As(V) respiratory reductase *arrA*, and the cytoplasmic detoxification reductase *arsC* were widespread. Reduction regulators (e.g., *arsR*, *arrR*, and *arrC*) were also present, consistent with operon-level regulation of Sb redox functions.

#### Detoxification and transport

MAGs harbored Sb(III) efflux and detoxification systems (e.g., *arsA*, *arsB*, *ACR2*, *ACR3, acrR*) likely involved in alleviating intracellular toxicity. Phosphate and glycerol phosphate transporters (*glpF, pit*, *pstABC*) were abundant and may facilitate Sb(III) and Sb(V) uptake due to its chemical analogy with phosphate. Resistance determinants (*arsH*, *arsP*, *arsO*, *pgpA*, *AS3MT*) and heavy-metal clusters (*czcABC*, *cus*, *cop*) indicate adaptation to metal-rich environments. Canonical photosynthesis markers (e.g., *psa/psb*, *puf/puh*, *bch/acs*) were not detected, supporting the non-phototrophic nature of these taxa.

#### Redox homeostasis and potential redox flexibility

Antioxidant defenses were ubiquitous, including glutathione metabolism genes (*gshA*, *gshB*, *gor*, *gst*), superoxide dismutase (*sod*), catalase (*katG*), and peroxidases (*ahpC*, *trx*, *grx*). These defense mechanisms likely maintain redox balance during sustained Sb(V) reduction. Notably, MAGs also encoded *aio*-type arsenite oxidase-like genes (*aioA*/*aioB*) and putative *anoA*-like genes, which is consistent with potential redox versatility that may limit intracellular Sb(III) excessive accumulation.

#### Electron transfer and energy supply

PESbR-associated MAGs contained multiple electron-transfer modules that may channel illumination-generated reducing power to terminal reductases, including cytochromes (*cyc*), DMSO reductase family genes (*dmsA*/*B*/*C/D*, *dmsR*), formate dehydrogenases (*fdhD*/*E*, *fdoG*/*H*), succinate/fumarate reductases (*sdh*/*frd*) and Ni–Fe hydrogenases (*hyaA*/*B*, *hydA*). Respiratory chain components were also prevalent, such as cytochrome oxidases (*coxA*/*B*/*CD*, *cydA*/*B*) and cytochrome *c* maturation proteins (*ccdA*, *ccmE*/*F*/*H*, *napC*, *nrfB*/*E*, *torC*/*Y*). Riboflavin biosynthesis genes (*ribB*, *ribBA*, *ribD*, *ribF*, *ribH*) were widespread, which suggests a potential role of FMN/FAD in intra- or respiratory electron transfer. Energy generation modules were abundant, including F-type ATP synthase subunits and multiple ABC transporters.

#### Respiratory diversification

Beyond Sb(V), PESbR MAGs encoded additional respiratory pathways, including marker genes associated with mercury, chromium, sulfate, and nitrate reduction. These pathways indicate potential competition among Sb(V) and different terminal electron acceptors for available photoelectrons under complex redox conditions.

#### Comparison with conventional Sb(V)‑reducer

To contrast the PESbR features (Fig. 4b), nine MAGs enriched in dark microcosms were considered as putative conventional reducers (Fig. 3e), including as *Rhodococcus* (bin.10, bin.3, bin.51), *Nocardioides* (bin.39), *Devosia* (bin.42, bin.29), and *Afipia* (bin.13). These genomes tended to contain fewer respiratory *anrA*/*arrA* but a higher prevalence of efflux pumps (*arsA/ACR*), resistance (*arsH/arsO*), and *aio*-type/anoA-like markers (*aioB/anoA*), which is consistent with a detoxification and export strategy rather than energy-conserving respiration.

### Metatranscriptomic validation of PESbR activity

**Fig. 5:**
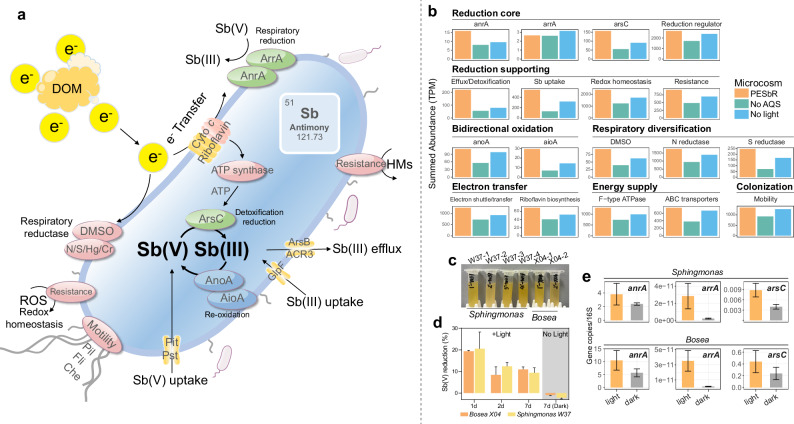
Multi-level evidence for photoelectron-assisted microbial Sb(V) reduction. **a** Schematic of the PESbR pathway showing the generation, transfer and utilization of photoelectrons for sustainable Sb(V) reduction. **b** Metatranscriptomic responses in PESbR microcosms relative to controls. **c** Isolation from sediments corresponding to two PESbR-associated genera, *Sphingomonas* and *Bosea*. **d** Sb(V) reduction by the isolates in pure-culture PESbR microcosms under light–dark cycles. **e** qPCR assays showing higher relative abundance of Sb(V) reduction-associated genes in light-exposed isolates compared with dark controls (16S-normalized). Data are presented as mean values ± SE.

Metatranscriptomic profiling further supported the potential coupling of photoelectron supply to Sb(V) reduction, together with enhanced Sb tolerance and redox-stress responses under PESbR conditions (Fig. 5a). Among > 3,800 detected transcripts (Supplementary Data 4), a large fraction (60–70%) were upregulated in PESbR microcosms compared to controls, indicating broad transcriptional activation (Fig. 5b). Core Sb(V) reduction genes and regulators responded strongly, including *anrA*, *arsC*, *arrR*/*arsR* increased by up to ~2.8 fold. While *arrA* transcript abundance remained similar to controls, possibly reflecting lower specificity for Sb(V) in an Sb-only system. Detoxification and uptake systems were also activated, including the efflux/transport genes (*arsA*, *arsB*, *acr3*) increased by up to ~4.4 fold; phosphate and glycerol-phosphate transporters (*pit* and *pst*) increased by up to ~6.8 fold, consistent with sustained Sb(V) uptake and Sb(III) export during reduction and detoxification. Notably, Sb(III) oxidation-related transcripts (*aioA* and *anoA*) were also upregulated by up to ~4.3 fold under reducing conditions, which is consistent with potential Sb(III)/Sb(V) redox versatility.

Metal resistance genes (*czcA*/C, *cusB*, *copA*, *zntA*) increased by up to ~8.9 fold. Antioxidant defenses (*sod*, *katG*, *ahpC*, *trx*, *grx*) increased by up to ~4.0 fold, consistent with mitigation of ROS damage under elevated photoelectron flux. Multiple alternative respiratory pathways also increased by up to ~4.6 fold, including nitrate and sulfate reduction, indicating the alternative photoelectron sinks were active alongside Sb(V). In line with this, addition of other terminal electron acceptors suppressed photoelectron-driven Sb(V) reduction (Supplementary Fig. 6), supporting competition among electron acceptors. Electron-transfer components were broadly upregulated by up to ~7.2 fold under PESbR conditions, including DMSO reductases, formate dehydrogenases, cytochrome oxidases, cytochrome biogenesis proteins, cytochrome complexes, Ni–Fe hydrogenases and riboflavin metabolism genes. Energy production modules (e.g., F-type ATP synthase, ABC transporters) increased by up to threefold, consistent with increased energy demand under PESbR conditions. Moreover, motility and colonization genes were also upregulated by several times, including pilus assembly (*pilA*, *cpaA*/*B*), flagella (*fliC*, *fliL*), chemotaxis (*cheD*/*W*/*X*/*Z*), and morphological flexibility (*flc*). These patterns may suggest active migration toward DOM-rich and light-exposed niches. Collectively, these data support a biologically mediated network linking Sb redox, stress defense, alternative respiration, electron transfer, energy production and motility under PESbR conditions.

### Physiological validation with PESbR isolates

To complement community-level evidence, we isolated native strains belonging to two key PESbR genera. Two representatives, *Sphingomonas* W37 and *Bosea* X04, were selected for validation (Fig. 5c). Both strains showed strong tolerance to Sb stress up to 2 mM (Supplementary Fig. 7). In anaerobic pure-culture PESbR microcosms, each strain reduced 10–30% of Sb(V) within 7 days (Fig. 5d). In parallel light–dark switching microcosms, Sb(V) reduction rates declined rapidly during dark periods (Supplementary Fig. 8), consistent with a light-dependent contribution. qPCR analyses further showed higher copy number of Sb-reducing genes in light-exposed cultures than in dark controls for both strains (Fig. 5e). The *anrA* increased 1.6–1.8 fold, *arrA* 26–27 fold, and *arsC* 1.9–2.2 fold. These results provide direct physiological evidence that indigenous sediment microorganisms can capture photoelectrons for Sb(V) reduction. Furthermore, comparative genomic analysis of public NCBI reference genomes of *Bosea* and *Sphingomonas* revealed that *anrA*, *arsC* and *arrA* are commonly present within these genera, suggesting that genetic potential relevant to PESbR may be widespread (Supplementary Fig. 9).

### Natural occurrence of PESbR potential in sediments

**Fig. 6:**
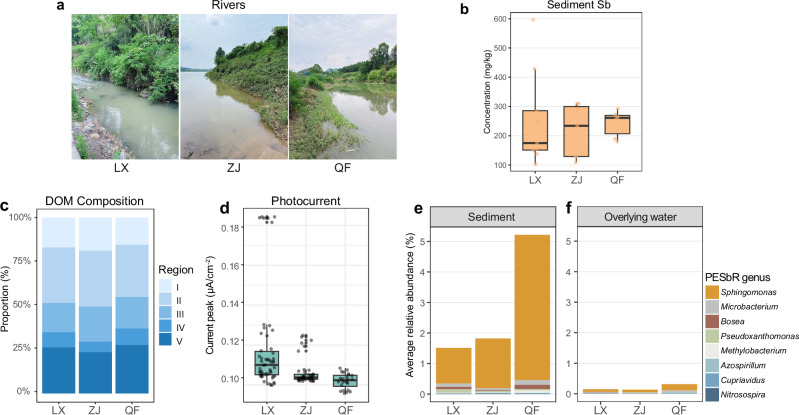
Field-scale survey of PESbR-related signatures in contaminated sediments. **a** Photos of sampling sites across the three major river systems near Xikuangshan (Lianxi-LX, Zijiang-ZJ, and Qingfeng-QF). **b** Sb concentrations in sediments. **c** DOM composition was resolved by 3D-EEM fluorescence regions: I (tyrosine-like), II (tryptophan-like), III (fulvic-like), IV (microbial by-product-like), and V (humic-like). **d** Photoelectrochemical activity of sediment-extracted DOM shown as photocurrent responses under illumination. The center line indicates the median; boxes show the 25th–75th percentiles; whiskers extend to 1.5× the interquartile range; points represent individual data. **e**, **f** Occurrence and relative abundance of major PESbR-associated genera in sediment **e** and overlying-water **f** communities.

To evaluate broader ecological prevalence, we surveyed contaminated sites along three river systems (LX, ZJ, QF, spanning ~270 km²) around Xikuangshan (Fig. 6a). Sediment Sb concentrations ranged from ~100–600 mg kg⁻¹ (Fig. 6b). 3D-EEM fluorescence (Supplementary Fig. 10) showed that sediment-extracted DOM ( ~ 20–530 mg DOC kg^-1^ dry weight) was dominated by humic-like (Region V; 24–28%) and fulvic-like (Region III; 17–20%) fractions (Fig. 6c). Fluorescence indices further suggest that DOM is predominantly freshly released (index >1) and highly humified (index ~1, Supplementary Fig. 11). All DOM extracts produced stable photocurrents ( ~ 0.10–0.18 *µ*A cm⁻²) under illumination, indicating that photosensitive DOM is common in these mine-impacted sediments (Fig. 6d). The corresponding photoelectron flux could, in principle, support an order-of-magnitude Sb(V) reduction potential of roughly 10–100 mg m⁻² d⁻¹ at illuminated sediment-water interface.

We detected eight of the ten core PESbR genera in paired sediments and overlying waters (Fig. 6e). These included *Azospirillum*, *Bosea*, *Cupriavidus*, *Methylobacterium*, *Microbacterium*, *Nitrosospira*, *Pseudoxanthomonas* and *Sphingomonas*, with *Sphingomonas* being the most abundant. These genera were more enriched in sediments (mean total ~2.36%) than in the overlying water column ( ~ 0.18%, Fig. 6f), suggesting habitat-specific niches and highlighting light-exposed surface sediments as potential hotspots for PESbR activity. Taken together, these results indicate that both photosensitive DOM signatures and PESbR-putative taxa are widespread in Sb-contaminated aquatic sediments, supporting broader environmental relevance of PESbR potentials.

## Discussion

Direct evidence that natural DOM mixtures^17,18^ in supporting microbial Sb(V) reduction has so far remained limited. Here we suggest that photosensitive DOM provides photoelectrons to sediment microorganisms and thereby support a Sb(V)-dependent photoelectrophy. This finding links DOM’s photoelectrochemical activity with the metabolic versatility of non-photosynthetic microbes and metal reduction in contaminated sediments. We first confirmed that several DOM types exhibited measurable photoelectrochemical activity, and that photocurrent depended on molecular composition, concentration and light intensity (Fig. 1). A model quinone mediator (i.e., AQS) can couple illumination-generated reducing power to microbial Sb(V) reduction in controlled PESbR microcosms (Fig. 2b). These indicate that DOM-derived photoelectrons can make a measurable contribution to Sb(V) bioreduction under experimental conditions. Because abiotic reductants may also convert Sb(V) to Sb(III)^18,27^, we further used dual-chamber setup to show that photoelectrons generated at the anode could drive Sb(V) reduction in the active biocathode (Fig. 2d), confirming the biological nature of PESbR rather than an abiotic photochemical process^26,27^. Besides, PESbR appears distinct from canonical phototrophy and engineered semiconductor-based biohybrids, which rely on intracellular photosystems in phototrophs or on solid minerals and engineered semiconductors as light absorbers^28^. Instead, non-phototrophic PESbR communities use photosensitive DOM as a dissolved light absorber that releases extracellular reducing power to drive Sb(V) reduction. This pattern may represent a more broadly distributed and naturally occurring metabolic mode in sediments.

We found that light and photosensitive DOM reshaped sediment microbial communities by reducing α-diversity and enriching ~ 60% PESbR-associated taxa (Fig. 3, Supplementary Fig. 5). Results also showed that these taxa collectively harbor Sb(V) reductase genes (*anrA*, *arrA*, *arsC* and their regulators), together with metal(loid)s and ROS resistance, and respiratory electron transfer modules (Figs. 4–5). Isolates belonging to *Sphingomonas* and *Bosea* also reduced Sb(V) using photoelectrons in pure culture, indicating that these indigenous taxa are capable of PESbR (Fig. 5c–e). Field surveys across mining-impacted river sediments further showed the co-occurrence of photosensitive DOM and PESbR-associated taxa (Fig. 6). These results suggest that PESbR is likely relevant in other Sb-contaminated sites as well. Notably, natural variability in DOM concentration and photoreactivity^21^, pH, light penetration depth^29^, suspended particles^30^, and hydrodynamics may affect PESbR activity. Future work will need to evaluate these environmental factors and their implications for Sb fate.

DOM is widespread in mining-impacted waters and sediments. PESbR shows that DOM is not only a carbon source or redox mediator in microbial metabolism but also acts as a natural photosensitizer that supports microbial Sb(V) reduction. DOM consists of diverse light-absorbing organic molecules^21^ and its photochemical potential relates to electron donating capacity^21^. Quinone-rich DOM fractions, which are widespread analogues of humic substances, generally show strong redox activity^17,31^ and therefore generate photoelectrons efficiently. Model quinones such as AQS have been shown to function as both microbial photosensitizer and capacitor in sustaining photocurrent generation even during alternating light and dark periods^32^. Sediment-extracted DOM in this study was dominated by humic-like components (24–28%, Fig. 6c). Even in certain ecosystems where biologically inert DOM predominates^33^, such DOM can still provide measured photoelectron flux under natural illumination. These photoelectrons may contribute to microbial metabolism and potentially influence Sb(V) reduction. A large-scale survey of river microbiomes further show that light-linked microbial energy metabolisms are common and tend to increase along hydrological gradients^34^. Taken together, these observations suggest that photosensitive DOM plays a central role in shaping sediment microbial communities and in mediating contaminant transformations under illuminated conditions.

PESbR contributes to Sb biogeochemical cycling and microbial photoelectrophy. Microbial processes are central to Sb speciation, mobility and bioavailability in sediments, yet this field remains less explored than the analogous As cycle^6,35^. To date, only about nine heterotrophic strains have been confirmed to reduce Sb(V)^7,36^. These organisms typically rely on organic substrates such as acetate, lactate, citrate or pyruvate^8^ to reduce Sb(V) through respiratory reductases AnrA^10^/ArrA^15^ or the detoxification enzyme ArsC^14^. Thus, in DOM-rich sediments, DOM has been regarded as a carbon and electron source for these heterotrophic pathways^3,7^. Our findings expand this understanding by suggesting that photoelectrons can enhance microbial Sb(V) reduction under controlled laboratory condition. In natural environments, the contribution of PESbR will likely vary with DOM composition, hydrodynamics and competition from alternative electron acceptors such as nitrate, sulfate, Fe(III), As(V), Cr(VI), Hg(II)^4,7^. In line with this, many PESbR taxa encode and upregulate multiple terminal reductases and electron-transfer modules (Figs. 4a and 5b), allowing the potential dynamic of photoelectrons flux among competing respiratory pathways.

PESbR also extends the concept of microbial photoelectrophy, in which non-phototrophic microbes utilize extracellular photoelectrons rather than relying on phototrophic energy capture or canonical chemolithotrophy^25,26^. Under PESbR conditions, specialist non-phototrophic taxa—including *Sphingomonas*, *Bosea*, *Cupriavidus*, *Achromobacter*, *Microbacterium*, and *Bradyrhizobium*—were enriched and showed photoelectron-assisted Sb(V) reduction traits. Most of them had not previously been characterized as Sb(V) reducer. For example, previous studies have mainly linked *Sphingomonas*^37^ and *Bosea*^38^ to Sb(III) oxidation, Sb resistance and plant-associated functions in Sb-contaminated habitats, whereas their roles in Sb(V) respiratory reduction or other dissimilatory metal(loid) reduction pathways have rarely been documented. Co-occurrence network analysis further revealed numerous positive associations between the major PESbR taxa and other community members (Supplementary Fig. 12), suggesting potential cooperation during Sb(V) reduction. Such interaction likely contributes to the higher Sb(V) reduction observed in mixed communities (Fig. 2b) compared with individual isolates (Fig. 5d).

Sb often coexists with As in contaminated sediments^39,40^, and both elements share several redox enzymes such as ArrA, ArsC and ArsR^4^. Yet, recent studies have revealed distinctions between two biogeochemical cycling^6^. For example, *Geobacter* sp. SVR selectively reduces Sb(V) without known As(V)-respiring capability (Yamamura et al. 2021), and Sb-specific enzymes like AnrA^10^, Sbr^16^, and AnoA^41^ have been reported. Our previous work on photoelectron-assisted As(V) reduction (PEAsR)^42^ identified a partial overlap between PESbR- and PEAsR-associated taxa (Supplementary Fig. 13, e.g., *Cupriavidus*, *Bradyrhizobium*, *Bosea*), suggesting shared extracellular electron utilization strategies and respiratory reductases during microbial photoelectrophy. In natural Sb–As co-contaminated sediments, DOM-derived photoelectrons are likely shared between Sb(V) and As(V) reduction, which reflect both the ubiquity of metal-dependent photoelectrophy and the distinct biogeochemical behaviors of these two analogues^4,6^.

PESbR contrasts with conventional Sb(V) reduction and concurrent Sb(III) oxidation. Comparative MAGs analyses indicate that PESbR-associated taxa encode more respiratory reductases (*anrA*) than conventional dark, heterotrophic Sb(III) reducers (Fig. 4b). These membrane or periplasm-localized enzymes are generally linked to energy-conserving Sb(V) respiration that directly couples extracellular electrons to Sb(V) reduction^36^. When photoelectron flux is high, microorganisms that possess ArrA alongside extracellular electron-transfer systems^43^ and ATP synthase therefore appear capture of using photoelectrons more efficiently under illuminated conditions (Fig. 5). A parallel case observed in sulfate-reducing bacteria, where direct uptake of extracellular photoelectrons can account for up to 90% of microbial sulfate reduction^44^. In contrast, when extracellular photoelectrons are limited, many heterotrophic bacteria may instead adopt cytoplasmic detoxification (ArsC)—where Sb(V) is reduced intracellularly and the resulting Sb(III) is exported through efflux pumps such as ACR3 or the ArsAB to mitigate toxicity and maintain cellular redox balance^4^. Previous work in a hydrogen-fed membrane biofilm reactor showed that Arr-mediated respiration and Ars-mediated detoxification can shift depending on Sb(V) input, in which *arrA* transcript levels increased with increasing Sb(V) flux until respiration was impaired, after which *arsC* expression sharply increased to compensate^14^. This switch from energy-conserving respiration to detoxification suggests that how the availability of extracellular photoelectrons shapes microbial Sb(V) reduction.

PESbR-associated taxa also expressed Sb(III) oxidation-related markers (*aioA*, *anoA*) even under reducing conditions (Figs. 4a and 5b). Pure-culture experiments confirmed that isolates such as *Sphingomonas* and *Bosea* can reduce Sb(V) (Fig. 5c–e), although these genera were previously known mainly for Sb(III) oxidation^38^. One possible interpretation is that these oxidation-associated markers reflect broader redox versatility in PESbR process. Under redox fluctuations or transient microoxic niches, AioA/AnoA may re-oxidize toxic Sb(III) produced during continuous Sb(V) reduction, thereby preventing intracellular accumulation of Sb(III), releasing excess reducing power, and maintaining cellular redox balance (Fig. 5a). Meanwhile, transient oxidants or microoxic niches generated by alternative electron acceptors (e.g., NO₃⁻, Fe(III) or sulfate) can persist in anoxic sediments^45^ and may sustain low-level activity of *aioA*/*anoA* in a standby state for rapid response to redox fluctuations. Similar coupling between anaerobic Sb(III) oxidation and nitrate reduction have been described elsewhere^46^. Moreover, recent study suggests that metal reduction can proceed under oxic exposure through flavin-mediated pathways^47^. This redox flexibility in PESbR resembles the survival strategy of nitrite oxidizers that thrive in ostensibly anoxic marine zones by using cryptic oxygen intrusions^48^. In sediments, frequent DOM pulses and hydrodynamic disturbances likely create transient microoxic niches^49^. These features suggest that PESbR microorganisms may alternate between Sb(V) respiration and Sb(III) oxidation depending on electron flux and local redox conditions. Such bidirectional flexibility could help explain microbial adaptation to dynamic and heterogeneous environments typically found in mining-impacted sediments.

Current knowledge of Sb(V) reduction remains limited, although this transformation can strongly influence Sb mobility in nature. This work suggests that in mine-impacted regions experiencing seasonal pulses of organic inputs, illumination of photosensitive DOM may promote biologically mediated Sb(V) reduction at anoxic sediment-water interfaces. Under sunny, low-flow conditions, elevated DOM concentrations and intense light could amplify both diel and seasonal Sb transformation. Models and risk assessments that treat DOM only as a carbon source or complexing agent might therefore underestimate its role in redox transformations. Conceptually, incorporating a “DOM-derived photoelectron flux” term could help capture this feature. Future work should quantify solid-phase Sb speciation and Sb(III) re-oxidation under redox fluctuations to assess the net implications of PESbR for Sb mobility and toxicity. In principle, interventions that alter DOM quality/concentration or light penetration may modulate Sb redox dynamics. Conversely, coupling controlled illumination with immobilization strategies (e.g., sulfide formation or iron-oxide co-precipitation) could help to retain reduced Sb(III) in situ, which may provide a conceptual basis for low-carbon treatment of mining water. Beyond Sb, PESbR-associated taxa encode diverse respiratory modules linked to arsenic, mercury, chromium, sulfur and nitrogen cycling, suggesting broader roles for photoelectrons in natural redox environments.

## Methods

### Study site and sediment collection

Field sampling was conducted at Xikuangshan (27°45′N, 111°49′E) in Lengshuijiang, Hunan Province. This subtropical mining region contains the world’s largest Sb deposit, with a mean annual temperature of 16.7 °C and 1,354 mm rainfall. Xikuangshan has experienced long-term Sb contamination in soil, groundwater, and nearby sediment^50^. Fresh surface sediments (0–10 cm) were collected along the near bank zone of mine-impacted streams. Samples were sieved (2 mm), homogenized, and transported at 4 °C for further analyses.

### Photoelectrochemical characterization of DOM

To assess the photoelectrochemical behavior, five types were selected to represent environmental variability^17^, including commercial humic acid and fulvic acid, mixed riverine DOM, sediment-derived DOM, together with the humic-like quinone model compound anthraquinone-2-sulfonate (AQS; 99.99 %, Aladdin Co.,)^31^. Each sample was dissolved to at environmentally relevant concentrations of 100–400 mg L⁻¹^51^. Photocurrent measurements employed a three-electrode cell connected to a CHI760E workstation, with a glassy-carbon working electrode (*Ø* 3 mm), graphite counter electrode and Ag/AgCl reference electrode^24^. Working electrodes were polished and sonicated sequentially in 1 M HNO₃, ethanol, and ultrapure water. A constant potential of +0.3 V versus Ag/AgCl was applied. Current–time (*I*–t) curves were recorded under light/dark cycles. Illumination was provided by a 300 W xenon lamp (PLS-SXE300 + ) equipped with an AM1.5 filter^24^. Illumination was calibrated with a light density meter and adjusted between 400–800 W m⁻² and lamp distance set at 2–10 cm to simulate natural variation^52^. 1 mM EDTA-2Na was added as a hole quencher for photocurrent assays only^42^ and was not added to any microcosm incubations.

### Batch PESbR microcosms

Single-chamber anaerobic PESbR microcosms (100 mL serum bottles) contained 90 mL minimal salts medium (pH=7.0), Sb(V) (0.2–1 mM as KSb(OH)₆), 5 mM photosensitizer (AQS used here) and 2 g fresh sediment. AQS was used as a defined quinone redox mediator (proxy for DOM quinone-like moieties in natural DOM) to isolate photoelectron transfer while minimizing DOM heterogeneity and carbon-substrate confounding. Bottles were flushed with N₂ (100 mL min⁻¹ for 30 min), sealed with butyl rubber stoppers and supplemented with resazurin ( ~ 4 *μ*M) as an anoxia indicator^53^. The indicator remained colorless throughout the incubations, indicating minimal oxygen intrusion. ORP remained stable with minor change during incubations. All incubations were incubated at 25 ± 1 °C under 12 h light/12 h dark cycles at 800 W m⁻², following illumination setting used for photocurrent assays.

Four controls were run in parallel with each lacking one component: no AQS; no light (maintained in foil-wrapped darkness); no added Sb(V); or no live biomass (sterilized sediment). All treatments were set up in triplicate. At regular intervals, 3 mL of supernatant was collected, filtered (0.22 *µ*m) and analyzed for Sb speciation (Sb(III), Sb(V), total Sb) by hydride-generation atomic fluorescence spectrophotometer (HG-AFS)^54^ with a recovery rate >90% and detection limit ( > 0.01 *μ*g L^-1^). Ar gas ( ≥ 99.999%) was used as the carrier gas (300 mL min^-1^) and shield gas (800 mL min^-1^). Given the high ratio of water-to-sediment (50:1) in the microcosm, solid-phase adsorption and release of Sb(V) were considered negligible.

### Photoelectron transfer assays

To decouple photoexcitation from direct microbial contact, a dual-chamber cell (100 mL per chamber) was separated by a Nafion 117 proton-exchange membrane (DuPont, USA)^24^. The photoanode chamber contained 5 mM photosensitizer (AQS) under illumination (800 W m⁻²), while the biocathode chamber contained 1 mM Sb(V) and sediment slurry in the dark, anaerobic conditions. Carbon cloth (7 cm × 7 cm) and indium tin oxide glass (4.5 cm × 4.5 cm) served as anode and cathode. Nafion membrane was pretreated with 5% H₂O₂ (80 °C, 1 h), deionized water soak (0.5 h), 5% H₂SO₄ boil (80 °C, 1 h) and a final deionized water rinse (0.5 h) before use. All reagents and illumination settings matched those used in batch microcosms. Sb(V) and Sb(III) concentrations in the biocathode chamber were periodically monitored.

### DNA/RNA extraction and high-throughput sequencing

Microcosms sediments were collected by centrifugation (5,000 × g, 5 min). DNA was extracted using the DNeasy PowerSoil Kit (Qiagen) followed with quality assessment^54^. RNA pellets were preserved in RNAprotect (Qiagen) and extracted using the RNeasy PowerSoil Kit followed by cDNA library construction. Metatranscriptomic reads were quantified as transcripts per million (TPM) followed by fold-change calculation. For 16S rRNA gene amplicons, the V3–V4 region was amplified with primers 341 F/806 R^55^. Purified amplicons were sequenced on Illumina MiSeq PE250 (Personalbio, Shanghai, China). Raw reads were processed in QIIME2 with DADA2 denoising^56^. The generated amplicon sequence variants (ASVs) were classified using SILVA v138^57^. Metagenomic libraries were sequenced on NovaSeq PE150, yielding ~20 GB per sample. Reads were assembled and binned into metagenome-assembled genomes (MAGs) using metaWRAP^58^. MAG quality was assessed with CheckM^59^. Taxonomy was assigned by GTDB-Tk^60^. Gene functions were annotated with KofamKOALA^61^. Sb-specific marker genes (*arrA*, *anrA*, *anoA*) were identified using custom HMMs^62^. Metatranscriptomic libraries were sequenced on an HiSeq 4000^53^. Adapter trimming and quality filtering were performed with Cutadapt v1.17^63^. Reads were assembled by Trinity^64^. MMseq2 removed redundant redundancy^65^, and TransGeneScan predicted coding sequences^66^. Transcript abundance was quantified as per million (TPM) using Salmon^67^.

### Isolation and validation of PESbR strains

A modified high-throughput cultivation protocol^68^ was used to effectively screen Sb-tolerant microbes from sediment slurries. Sediment slurries were subjected to ~10⁶-fold limiting dilution into multiple 96-well plates supplemented with 0.1 mM Sb stress to obtain single-cell inocula, followed by screening of turbid wells for PESbR activity. Candidate isolates were identified by 16S rRNA gene sequencing. Six isolates belonging to two key PESbR genera (*Sphingomonas* and *Bosea*) were obtained. Each isolate was then incubated in 100 mL anaerobic Sb(V)-reducing microcosms containing 0.5 mM Sb(V) and 5 mM photosensitizer under 12 h light/12 h dark cycles (as above). Sb(V) reduction after 7 days was quantified by AFS. Abundances of Sb(V)-reducing genes (*anrA*, *arrA*, *arsC*) were quantified by qPCR^68^ (primers listed in Supplementary Table 1).

### Cross-validation of PESbR potential in natural sediments

Paired sediments and overlying waters were collected along contamination gradients from three major river systems (Lianxi (LX), Zijiang (ZJ), and Qingfeng (QF) rivers, spanning ~270 km²) near Xikuangshan^50^. DOM in sediments was extracted from freeze-dried samples using solid-phase resin. Water samples were filtered at 0.22 *µ*m to obtain dissolved fractions^21^. DOM fraction was profiled by three-dimensional excitation–emission matrix (3D-EEM) fluorescence spectroscopy. Five regions were identified using established excitation/emission ranges^69^: Region I (tyrosine-like), Region II (tryptophan-like), Region III (fulvic-like), Region IV (microbial by-product-like), and Region V (humic-like). Relative contribution of each fraction was calculated as integrated areas normalized to total fluorescence. Sb concentrations in sediments, photoelectrochemical activity of DOM extracts, and microbial communities were measured as described above.

### Statistical analysis

ASVs abundances were normalized to relative abundances prior to statistical analyses. Microbial composition and α/β diversity were analyzed in MicrobiomeAnalyst using normalized ASV datasets^70^. Differentially abundant taxa were identified using LEfSe, with a Kruskal–Wallis test (*p* < 0.05) followed by linear discriminant analysis (LDA) to estimate effect size (LDA score > 2.0). Other data analyses were performed in GraphPad Prism and R. Statistical significance was determined by one-way ANOVA with Tukey’s post hoc test.

### Reporting summary

Further information on research design is available in the Nature Portfolio Reporting Summary linked to this article.

## Supplementary information

Supplementary Information

Peer Review File

Description of Additional Supplementary Files

Supplementary Data 1

Supplementary Data 2

Supplementary Data 3

Supplementary Data 4

Reporting Summary

## Source data

Source data

## Data Availability

The authors declare that all data supporting the findings of this study are available in the article and its Supplementary Information (including Supplementary Fig. 1−13 and Supplementary Table 1). Source data are provided with this paper. Sequencing datasets for 16S rRNA amplicons, metagenomics and metatranscriptomics have been deposited in NCBI under accession number PRJNA1309294, PRJNA1309780, and PRJNA1310047, respectively. Source data are provided with this paper.
