# Supplementary material for: Dissolved organic matter-generated photoelectrons enable microbial antimonate reduction in mine stream sediments
Source: Nat Commun. 2026 Apr 20;17:5463. doi: 10.1038/s41467-026-72108-1 (PMC13284237; doi:10.1038/s41467-026-72108-1)
Supplement: Supplementary file 1 — Supplementary Information [file 41467_2026_72108_MOESM1_ESM.pdf]

Supplementary Information

Dissolved organic matter-generated photoelectrons enable microbial antimonate reduction in mine stream sediments

Linao Zhu [1, †], Hanbing Gao [1, †], Min Shen [2], Kuanxin Huang [2], Yongqun Tang [2], Lele He [3], Jing Huang [4], Cheng Zhao [1], Weiping Xiong [3], Xiaoli Ni [1], Honghui Wu [1], Shikai Li [1], Zhaohui Guo [1], Jie Cao [1], Wenjing Xue [5], Rui Xu [1, *]

[1] Institute of Environmental Engineering, School of Metallurgy and Environment, Central South University, Changsha 410083, P.R. China

[2] Urban Geological Survey and Monitor Institute of Hunan Province, Changsha, 410007, P.R. China

[3] College of Environmental Science and Engineering, Hunan University, Changsha 410012, P.R. China

[4] State Key Laboratory of Woody Oil Resources Utilization, Hunan Academy of Forestry, Changsha 410004, P.R. China

[5] College of Environmental Science and Engineering, Yangzhou University, Yangzhou 225009, P.R. China

[†] These authors contributed equally: Linao Zhu and Hanbing Gao.

[*] Corresponding author:
Dr. Rui Xu
Associate Professor
Email: xurui7@csu.edu.cn
No. 932 Lushan South Road, Changsha 410083, PR China

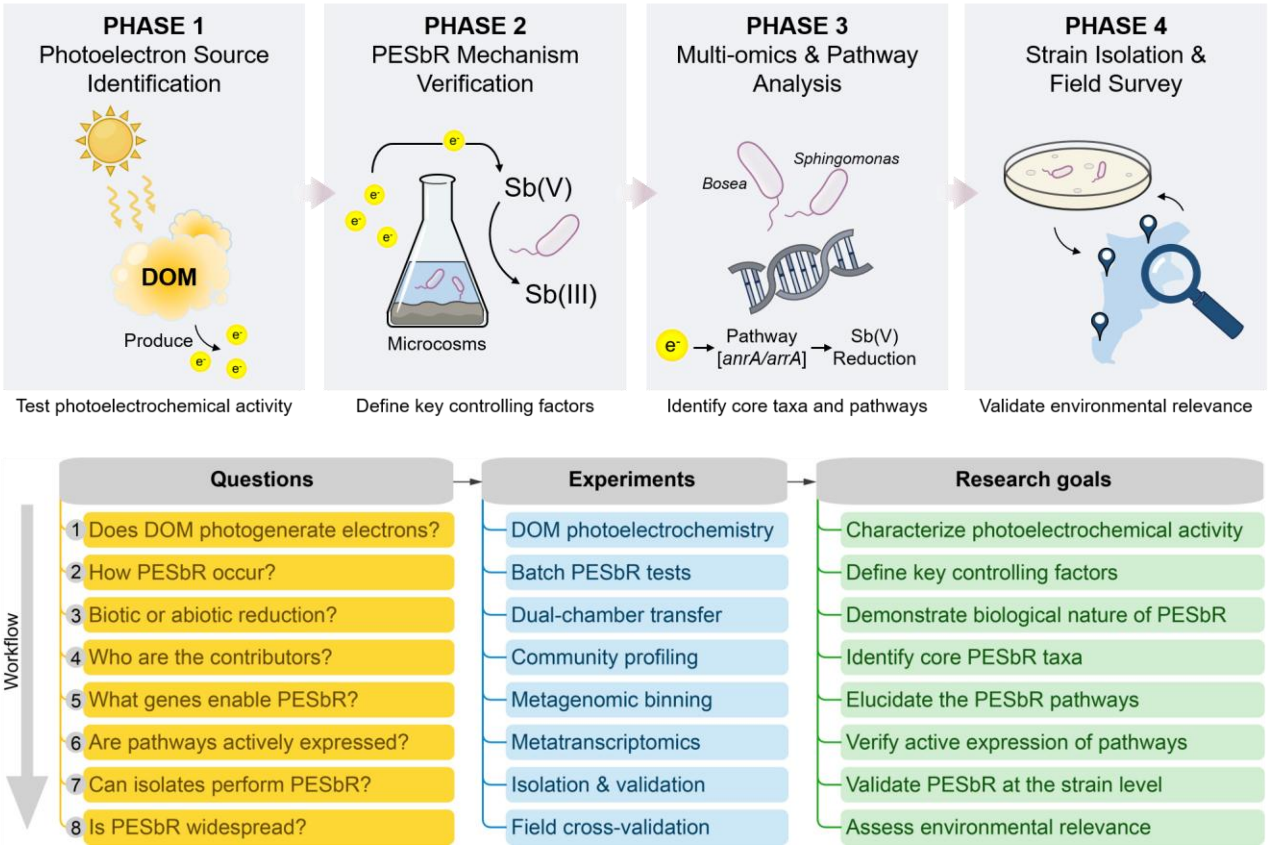

Supplementary Fig. 1 Study workflow demonstrating that DOM-generated photoelectrons enable microbial Sb(V) reduction in mine-stream sediments. Steps include DOM photoelectrochemical assays, batch and dual-chamber microcosms, multi-omics profiling, isolation and testing of PESbR strains, and field-scale cross-validation.

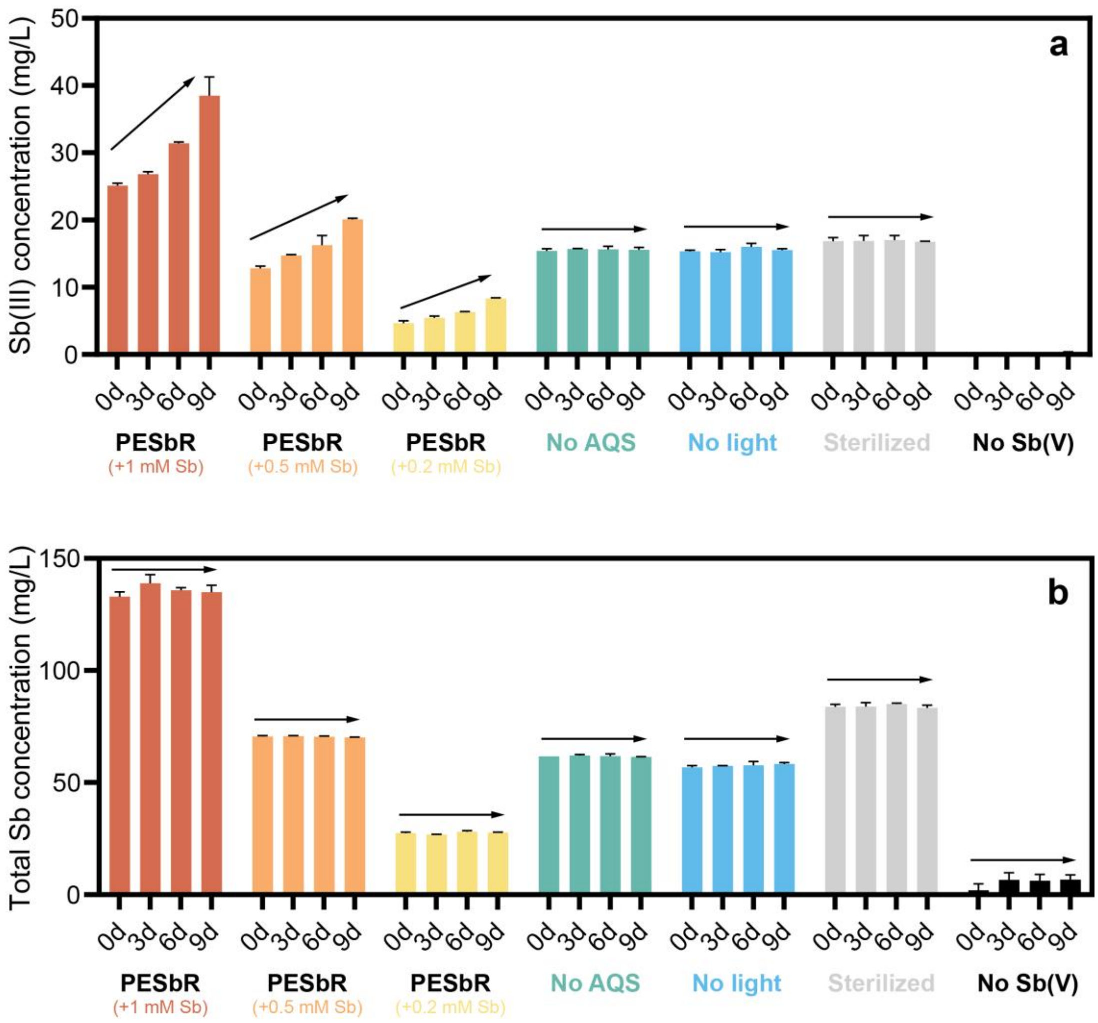

Supplementary Fig. 2 Supplementary data for Sb(V) reduction in batch microcosms. (a) Only the complete PESbR microcosm (light + AQS + Sb(V) + live biomass) shows substantial Sb(III) accumulation over time, whereas controls lacking any single component (no AQS, no light, no added Sb(V), or sterile sediment) show negligible change. (b) Total Sb concentration remains constant in all microcosms over time to demonstrate mass balance.

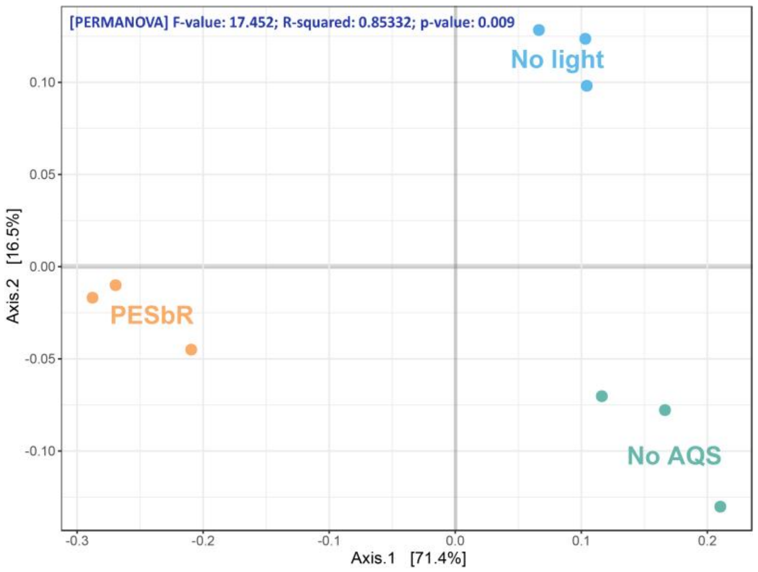

Supplementary Fig. 3 Microbial β-diversity based on Bray–Curtis distances for PESbR microcosms versus controls without light or without AQS.

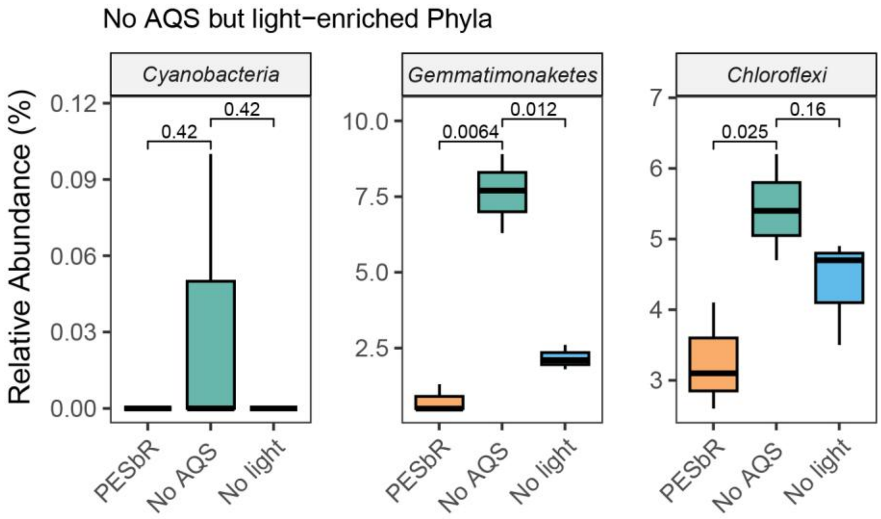

Supplementary Fig. 4 Relative abundances of phototrophic taxa specifically enriched in the light only and AQS-free control microcosms.

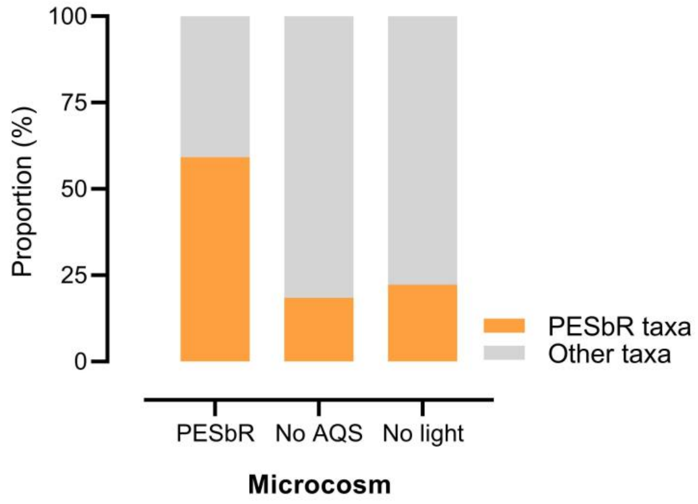

Supplementary Fig. 5 Proportion of PESbR-associated taxa versus other taxa across microcosm treatments. Bars show the summed relative abundance of all PESbR-associated genera (defined by LEfSe, LDA > 2; Supplementary Data 1) and all remaining genera (“other taxa”).

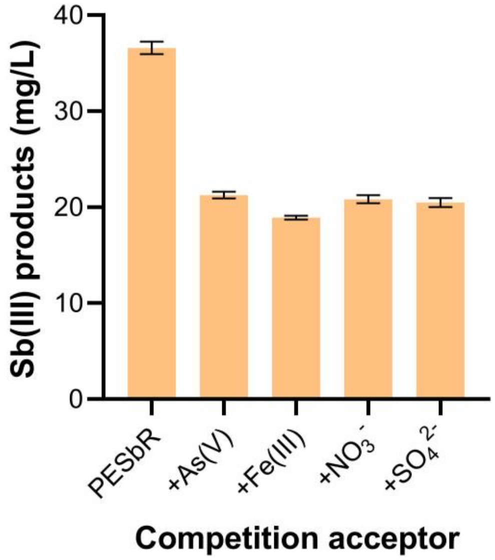

Supplementary Fig. 6 The addition of alternative terminal electron acceptors suppresses photoelectron-driven Sb(V) reduction, as reflected by the decreased Sb(III) products.

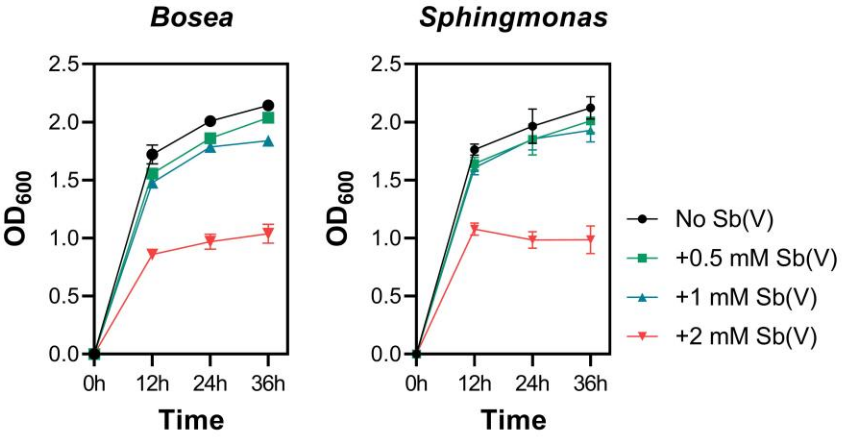

Supplementary Fig. 7 Growth of *Bosea* and *Sphingomonas* strains under different Sb(V) stress.

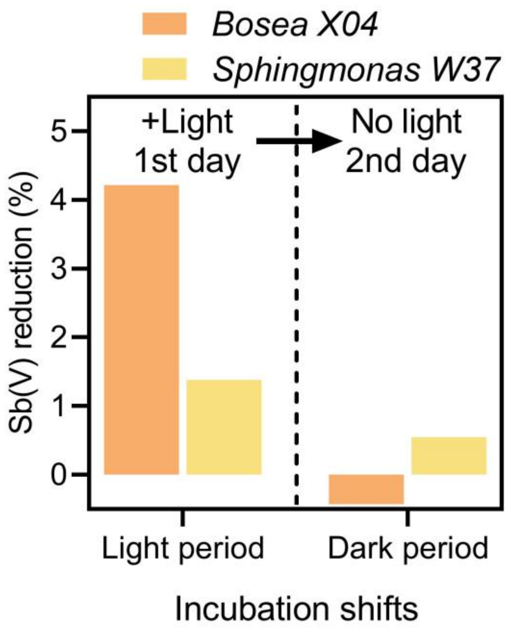

Supplementary Fig. 8 Sb(V) reduction dynamics in time-resolved light–dark switching microcosms.

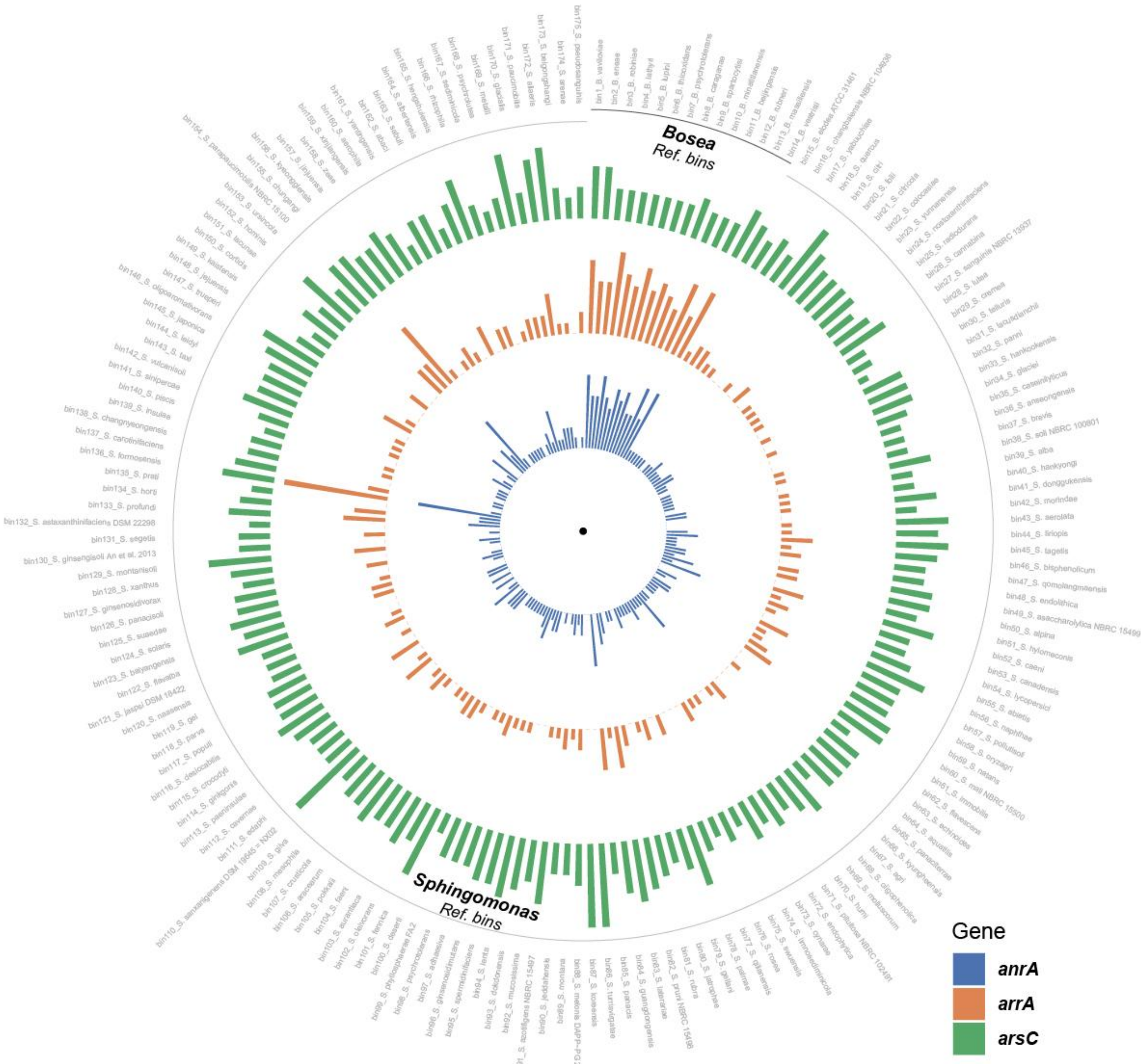

Supplementary Fig. 9 Comparative genomic analysis of public NCBI reference genomes from two PESbR-enriched genera (*Bosea* and *Sphingomonas*) shows gene repertoires broadly consistent with the recovered MAGs (e.g., *anrA*/*arsC*/*arrA*), suggesting that genetic potential for Sb redox transformations may be common within these genera.

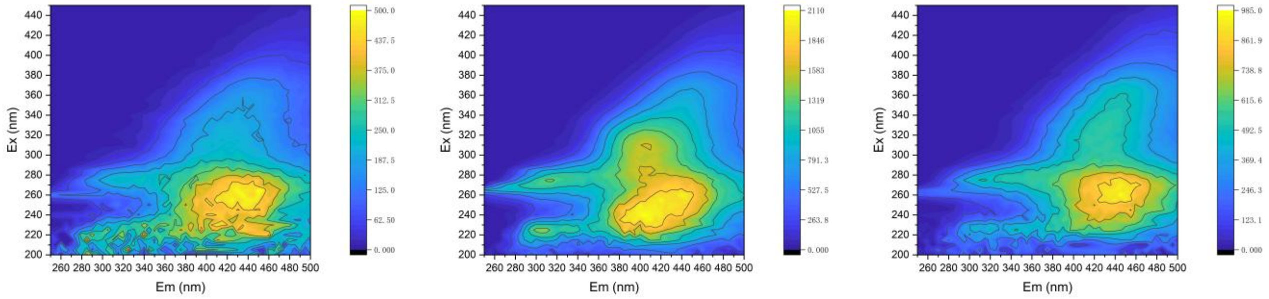

LX ZJ QF

Supplementary Fig. 10 3D-EEM fluorescence of sediment-extracted DOM from the three major river systems around Xikuangshan: Lianxi (LX), Zijiang (ZJ), and Qingfeng (QF) rivers.

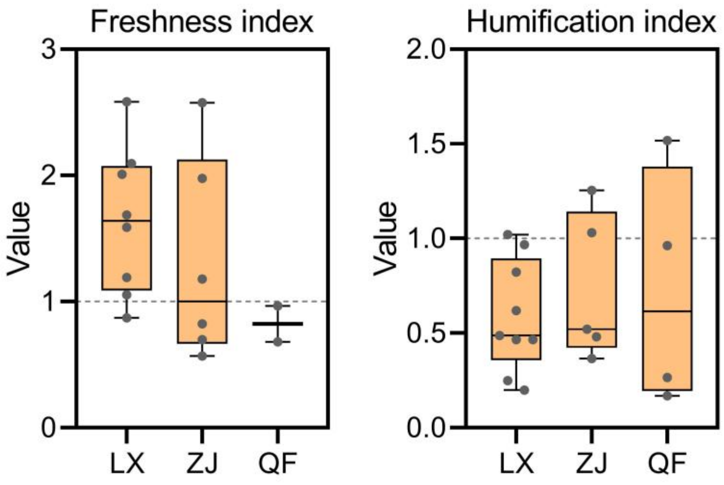

Supplementary Fig. 11 Fluorescence index–based characterization of DOM putative sources and compositional properties, using the freshness index (FI) and humification index (HIX).

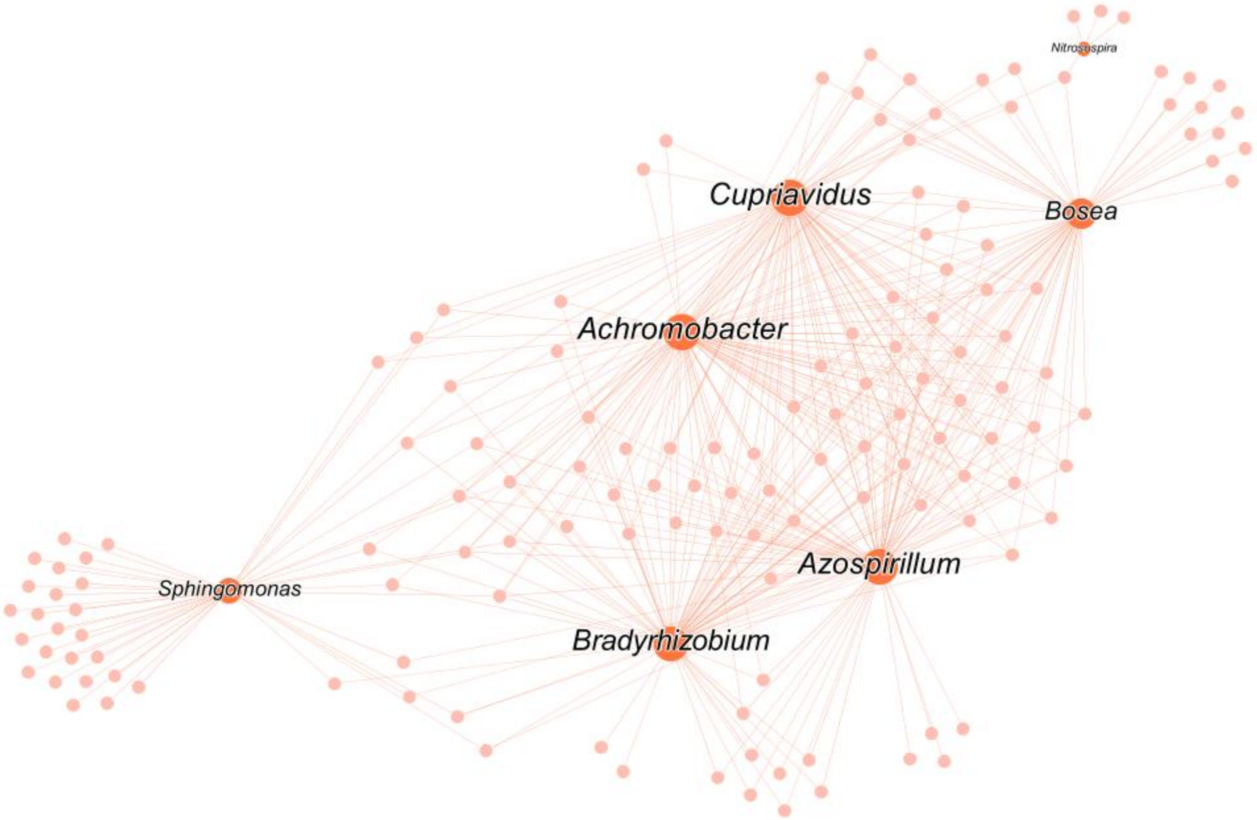

Supplementary Fig. 12 Co-occurrence network showing numerous positive links between main PESbR taxa (orange nodes) and other community members (pink nodes).

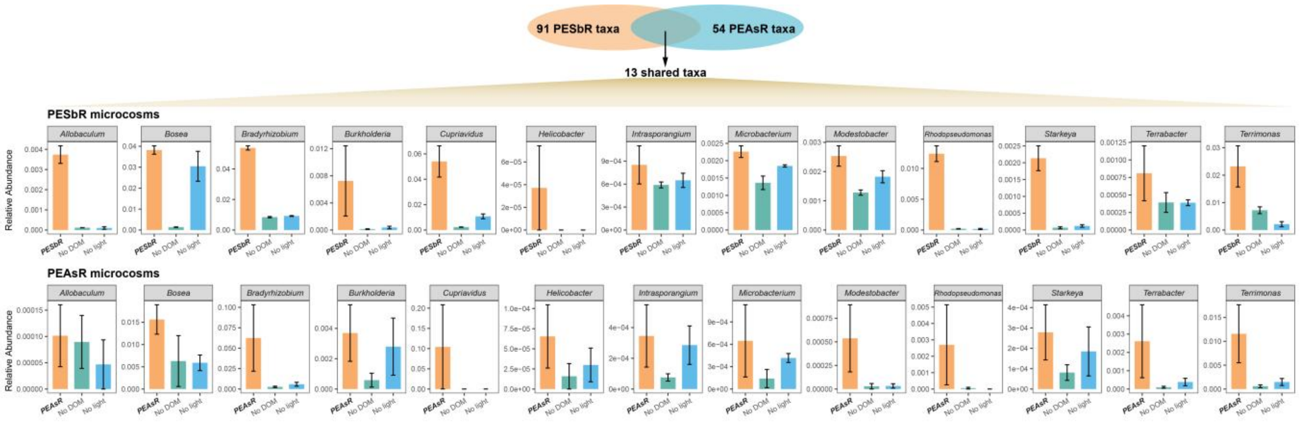

Supplementary Fig. 13 Cross-comparison of Sb(V) and As(V) as acceptors of photoelectrons under similar microcosm conditions. Venn diagram and relative abundance showing taxonomic overlap within putative PESbR genera (Sb(V)) and putative PEAsR genera (As(V)).

Supplementary Table 1 Quantitative PCR (qPCR) assays used to evaluate Sb(V)-reducing capacity of isolates.

Gene	16S rRNA	*arrA*	*arsC*	*anrA*
Primers	341F<br>518R	arrA-CVF1<br>arrA-CVR1	amlt−42F<br>amlt−376R	Desul-anrAF<br>Desul-anrAR
Sequences	TACGGGAGGCAGCAG<br>ATTACCGCGGCTGCTGG	CACAGCGCCATCTGCGCCGA<br>CCGACGAACTCCYTGYTCCA	TCGCGTAATACGCTGGAGAT<br>ACTTTCTCGCCGTCTTCCTT	GAAAAACAGCTACGCGGTGG<br>CTGGGTGTGTATTTCCGGCT
Thermal protocol	95ºC --> 15 min<br>**95ºC --> 10 sec**<br>**55ºC --> 30 sec**<br>**Plate Read**<br>Melt Curve 65.0°C to 95.0°C: Increment 0.5°C<br>#Steps in **bold × 40 cycles**	95ºC --> 15 min<br>**95ºC --> 10 sec**<br>**60ºC --> 30 sec**<br>**Plate Read**<br>Melt Curve 65.0°C to 95.0°C: Increment 0.5°C<br>#Steps in **bold × 40 cycles**	95ºC --> 15 min<br>**95ºC --> 10 sec**<br>**56ºC --> 30 sec**<br>**Plate Read**<br>Melt Curve 65.0°C to 95.0°C: Increment 0.5°C<br>#Steps in **bold × 40 cycles**	95ºC --> 15 min<br>**94ºC --> 45 sec**<br>**60ºC --> 45 sec**<br>**Plate Read**<br>Melt Curve 65.0°C to 95.0°C: Increment 0.5°C<br>#Steps in **bold × 40 cycles**
Standard curves R^2	0.992	0.992	0.994	0.990
