# Supplementary material for: Dissolved organic matter-generated photoelectrons enable microbial antimonate reduction in mine stream sediments
Source: Nat Commun. 2026 Apr 20;17:5463. doi: 10.1038/s41467-026-72108-1 (PMC13284237; doi:10.1038/s41467-026-72108-1)
Supplement: Supplementary file 3 — Description of Additional Supplementary Files [file 41467_2026_72108_MOESM3_ESM.pdf]

Description of Additional Supplementary Files

Supplementary Data 1: LEfSe-identified taxa enriched in PESbR and no-light controls

Supplementary Data 2: Summary statistics for recovered PESbR MAGs

Supplementary Data 3: Presence of PESbR-related genes in MAGs

Supplementary Data 4: Metatranscriptomic abundances (TPM) for key PESbR-related genes
