# Supplementary material for: Dissolved organic matter-generated photoelectrons enable microbial antimonate reduction in mine stream sediments
Source: Nat Commun. 2026 Apr 20;17:5463. doi: 10.1038/s41467-026-72108-1 (PMC13284237; doi:10.1038/s41467-026-72108-1)
Supplement: Supplementary file 8 — Reporting Summary [file 41467_2026_72108_MOESM8_ESM.pdf]

nature portfolio

Corresponding author(s): Rui Xu

Last updated by author(s): Feb 21, 2026

Reporting Summary

Nature Portfolio wishes to improve the reproducibility of the work that we publish. This form provides structure for consistency and transparency in reporting. For further information on Nature Portfolio policies, see our Editorial Policies and the Editorial Policy Checklist.

Statistics

For all statistical analyses, confirm that the following items are present in the figure legend, table legend, main text, or Methods section.

n/a	Confirmed	
☐	☒	The exact sample size (*n*) for each experimental group/condition, given as a discrete number and unit of measurement
☐	☒	A statement on whether measurements were taken from distinct samples or whether the same sample was measured repeatedly
☐	☒	The statistical test(s) used AND whether they are one- or two-sided<br>*Only common tests should be described solely by name; describe more complex techniques in the Methods section.*
☒	☐	A description of all covariates tested
☒	☐	A description of any assumptions or corrections, such as tests of normality and adjustment for multiple comparisons
☐	☒	A full description of the statistical parameters including central tendency (e.g. means) or other basic estimates (e.g. regression coefficient) AND variation (e.g. standard deviation) or associated estimates of uncertainty (e.g. confidence intervals)
☒	☐	For null hypothesis testing, the test statistic (e.g. *F*, *t*, *r*) with confidence intervals, effect sizes, degrees of freedom and *P* value noted<br>*Give P values as exact values whenever suitable.*
☒	☐	For Bayesian analysis, information on the choice of priors and Markov chain Monte Carlo settings
☒	☐	For hierarchical and complex designs, identification of the appropriate level for tests and full reporting of outcomes
☒	☐	Estimates of effect sizes (e.g. Cohen's *d*, Pearson's *r*), indicating how they were calculated

Our web collection on statistics for biologists contains articles on many of the points above.

Software and code

Policy information about availability of computer code

Data collection	Data collection was performed using standard commercial instrument software, such as the electrochemical workstation software and manufacturer-provided software for sequencing and analytical instruments. No custom code was used for data collection.
Data analysis	Data analysis was performed using established open-source and commercial software, including QIIME2 with DADA2 for amplicon sequence analysis, metaWRAP, GTDB-Tk and CheckM for metagenomic analysis, Trinity and Salmon for metatranscriptomic analysis, MicrobiomeAnalyst for community statistics, and R and GraphPad Prism for data processing and visualization. No custom code or unpublished algorithms were used in this study.

For manuscripts utilizing custom algorithms or software that are central to the research but not yet described in published literature, software must be made available to editors and reviewers. We strongly encourage code deposition in a community repository (e.g. GitHub). See the Nature Portfolio guidelines for submitting code & software for further information.

Data

Policy information about availability of data

All manuscripts must include a data availability statement. This statement should provide the following information, where applicable:

- Accession codes, unique identifiers, or web links for publicly available datasets
- A description of any restrictions on data availability
- For clinical datasets or third party data, please ensure that the statement adheres to our policy

All data supporting the findings of this study are available within the Article and its Supplementary Information. Source Data are provided with this paper. Sequencing data have been deposited in the NCBI BioProject database under accession numbers PRJNA1309294 (16S rRNA gene amplicons), PRJNA1309780 (metagenomes) and PRJNA1310047 (metatranscriptomes).

Research involving human participants, their data, or biological material

Policy information about studies with human participants or human data. See also policy information about sex, gender (identity/presentation), and sexual orientation and race, ethnicity and racism.

Reporting on sex and gender	This study did not involve human participants, human data, or human biological materials; therefore, considerations of sex and gender are not applicable.
Reporting on race, ethnicity, or other socially relevant groupings	This study did not involve human participants or socially relevant groupings such as race or ethnicity.
Population characteristics	No human population characteristics are applicable, as this study did not involve human participants.
Recruitment	No participant recruitment was performed, as this study did not involve human participants.
Ethics oversight	Ethics approval was not required for this study, as it did not involve human participants, human data, or human biological materials.

Note that full information on the approval of the study protocol must also be provided in the manuscript.

Field-specific reporting

Please select the one below that is the best fit for your research. If you are not sure, read the appropriate sections before making your selection.

☐ Life sciences ☐ Behavioural & social sciences ☒ Ecological, evolutionary & environmental sciences

For a reference copy of the document with all sections, see nature.com/documents/nr-reporting-summary-flat.pdf

Ecological, evolutionary & environmental sciences study design

All studies must disclose on these points even when the disclosure is negative.

Study description	This study combined controlled sediment microcosm experiments with field surveys to investigate photoelectron-assisted microbial antimonate reduction. Laboratory experiments used factorial microcosm designs manipulating light exposure, dissolved organic matter, antimonate, and microbial activity, with biological replicates for each treatment. Multi-omics analyses, physiological validation, and filed survey were integrated to link geochemical processes with microbial community responses.
Research sample	The research samples consisted of surface sediments (e.g., 0-10 cm) collected from mining-impacted river systems in the Xikuangshan antimony mining region, China. These sediments were selected to represent antimony-contaminated, organic-matter-rich aquatic environments where microbial redox processes are environmentally relevant.
Sampling strategy	Sediment samples were collected using established environmental sampling procedures to cover upstream–downstream gradients and a broad range of river systems. Sample sizes were determined based on site accessibility, sediment availability, and consistency with prior studies of sediment microcosms and microbial redox processes. No formal statistical sample size calculation was performed.
Data collection	Data were collected by the research team following standardized experimental and analytical protocols. Geochemical measurements, microbial community analyses, and sequencing data were generated using established laboratory methods and commercial instrumentation as described in the Methods section.
Timing and spatial scale	Field samples were collected during discrete sampling campaigns (in 2024) from multiple river systems within the Xikuangshan mining region, spanning an area of approximately 270 km^2. Laboratory microcosm experiments were conducted over days to weeks under controlled conditions to capture microbial and geochemical dynamics.

Data exclusions	No data were excluded from the analyses.
Reproducibility	Reproducibility was ensured through the biological replicates, independent experimental repeats, and complementary analytical approaches.
Randomization	Randomization was not performed because microcosms were assembled under controlled laboratory conditions and treatments were applied according to a predefined experimental design.
Blinding	Blinding was not implemented because data acquisition and analysis were based on instrumental measurements and computational analyses, and therefore were not subject to subjective interpretation.

Did the study involve field work? ☒ Yes ☐ No

Field work, collection and transport

Field conditions	Field sampling was conducted in a subtropical mining region characterized by a mean annual temperature of approximately 16.7 °C and an average annual rainfall of about 1,354 mm. Sampling was performed under typical ambient field conditions for the region.
Location	Sediment samples were collected from mining-impacted river systems in the Xikuangshan antimony mining area, Lengshuijiang, Hunan Province, China (approximately 27°45′ N, 111°49′ E).
Access & import/export	All field sampling was conducted in compliance with local and national regulations. No special permits were required, and no protected areas or restricted species were involved. Samples were collected and transported within China for laboratory analysis.
Disturbance	Field sampling caused minimal disturbance, as only small amounts of surface sediment were collected. Sampling was conducted carefully to minimize disruption to the surrounding environment.

Reporting for specific materials, systems and methods

We require information from authors about some types of materials, experimental systems and methods used in many studies. Here, indicate whether each material, system or method listed is relevant to your study. If you are not sure if a list item applies to your research, read the appropriate section before selecting a response.

Materials & experimental systems

n/a	Involved in the study	
☒	☐	Antibodies
☒	☐	Eukaryotic cell lines
☒	☐	Palaeontology and archaeology
☒	☐	Animals and other organisms
☒	☐	Clinical data
☒	☐	Dual use research of concern
☒	☐	Plants

Methods

n/a	Involved in the study	
☒	☐	ChIP-seq
☒	☐	Flow cytometry
☒	☐	MRI-based neuroimaging

Plants

Seed stocks	This study did not involve the use of plants, seeds, or other plant materials.
Novel plant genotypes	No novel plant genotypes were generated or used in this study.
Authentication	Authentication procedures were not applicable, as no plant materials were used in this study.
