# Supplementary material for: Dissolved organic matter-generated photoelectrons enable microbial antimonate reduction in mine stream sediments
Source: Nat Commun. 2026 Apr 20;17:5463. doi: 10.1038/s41467-026-72108-1 (PMC13284237; doi:10.1038/s41467-026-72108-1)
Supplement: Supplementary file 2 — Peer Review File [file 41467_2026_72108_MOESM2_ESM.pdf]

nature portfolio

Peer Review File

Dissolved organic matter-generated photoelectrons enable microbial antimonate reduction in mine stream sediments

Corresponding Author: Dr Rui Xu

This file contains all reviewer reports in order by version, followed by all author rebuttals in order by version.

Version 0:

Reviewer comments:

Reviewer #1

(Remarks to the Author)
Microbial reduction of Sb(V) is a crucial factor in the migration of sb in mine stream sediments. This process is dependent on heterotrophic microorganisms that utilize dissolved organic matter as the carbon source. This manuscript reports a novel and environmentally significant mechanism of Sb(V) reduction mediated by non-phototrophic microorganisms utilizing photoelectrons generated from photosensitive dissolved organic matter (DOM) in sediments. This challenges the traditional understanding that Sb(V) reduction in sediments is exclusively driven by heterotrophic respiration using DOM as a carbon source. The authors adopted a series of advanced framework to support this new conceptual PESbR model. Authors demonstrate that light-excited DOM stimulates the activity of multiple non-phototrophic genera and associated redox genes, leading to enhanced Sb(V) reduction. These findings are interesting and carry significant environmental implications. The objectives of this study are clear. However, several key issues must be addressed to strengthen the clarity, environmental relevance, and broader applicability of the findings.

Major comments:
The use of $K_2Sb_2(C_4H_2O_6)_2$ may introduce organic tartrate, which is a readily metabolizable carbon and electron source for a wide range of heterotrophs. This complicates the interpretation that photoelectrons from DOM are the sole drivers of Sb(V) reduction. Recommend either replace with inorganic $KSb(OH)_6$ or include a tartrate-only control.

Supporting data for electron competition effects are lacking. Despite electron acceptor competition is briefly discussed in this manuscript, Sb is rarely the sole terminal electron acceptor in sediments. Please provide common potential acceptor assays, this would help confirm Sb(V)'s priority.

Although three rivers were tested for PESbR occurrence, but all are close to Xikuangshan. Readers will wonder if PESbR is specific to this area. Could the authors include additional representative Sb-contaminated rivers or conduct a wider comparison to increase the environmental relevance? This would increase the ecological scope.

The manuscript should better discuss the key environmental variables that modulate PESbR, such as pH, seasonal DOM pulses, sediment optical depth, light penetration (turbidity, shading), algal blooms, and suspended particles. Identifying the conditions in which PESbR is favored or constrained would help indicate its real-world impact and inform models or remediation strategies.

DOM is reported in mg L^{-1} in the electrochemical section and in mM in microcosm incubations, which is confusing. Please standardize it across all sections. This will allow comparison with environmental conditions.

For the microcosm light intensity, how was the light intensity chosen? Does it reflect natural irradiance levels in sediment–water interfaces? Please justify the representativeness of light settings. Also, whether the high-intensity near-source illumination (e.g., 800 W m^{-2} at 2 cm) could have introduced thermal artifacts or oxygen intrusion?

PESbR is described as distinct from semiconductor-driven or phototrophic reduction. Try to clarify this to prior known studies particularly in terms of microbial phototrophy or electroactive processes.

Current isolate experiments show low reduction (~10–30%), but no dark controls are provided. Proving these strains exhibit

PESbR needs stronger evidence. Additionally, the statement that reduction rates are comparable to heterotrophic respiration is misleading—heterotrophic controls reportedly reach >90%. In addition, try to include cell growth or Sb tolerance for these strains.

The difference of reduction rates between isolates and communities is intriguing. The superior performance of mixed communities over isolates suggests the presence of microbial cooperation. It is suggested to consider possible co-occurrence or interaction involving Pseudomonas and Sphingomonas and other “helper strains” in detoxification, or EET facilitation or cofactors.

The comparison of anrA and arrA genes involved in PESbR pathway is important. Discuss the functional differences between two pathways in Sb(V) reduction and whether they are associated with DOM.

The reported photoelectron current from field DOM are valuable. How to correlate such electron generation with estimated Sb(V) reduction potential?

The manuscript does not clearly distinguish whether Sb(III) is present in dissolved, sorbed, or mineralized forms. Suggest adding data to determine the speciation and environmental fate of reduced Sb. This will help evaluate the remediation potential of PESbR.

PESbR is mainly proposed to occur in sediments, but how deep can light penetrate? Sediments may do not receive much incident sunlight. Could this process be occurred in overlying waters? Try to consider the depth limitations in natural systems.

Figure. 3 clearly shows the distinct members responsible for different microcosms, but why not show taxa enriched in no DOM (light-only) controls? These could represent members prefer to light. Including this would help identify phototroph-enriched but PESbR-independent taxa.

Fig. 6: Try to provide DOM classification with more informative analysis beyond the simple 3D-EEM clusters.

It would help the reader to include a schematic overview of the full experimental workflow (currently missing in Figure S1).

How was light calibration performed?

How were Rayleigh scatters removed in EEM data?

Why was EDTA-2Na chosen as the quencher, and what are its potential effects on DOM behavior?

Need more discussion and comparison of Pseudomonas and Sphingomonas' role in Sb reduction. Current statement is rough.

Please clarify how PESbR strains were “efficiently” isolated from sediment slurry.

It is suggested to provide quantitative rate data (μmol Sb L^{-1} h^{-1}) in addition to % efficiency.

The manuscript would benefit from a clearer discussion of the practical implications of PESbR.

In the dual-chamber systems, the reduction rate of Sb(V) at the biocathode was relatively low compared to batch microcosms. Why? Which factors may hinder the efficiency?

Unify terminology throughout the maintext.

Fix unit formatting (μA cm^{-2}, W m^{-2}, mg C L^{-1}).

Correct coordinates formatting (27°78′ N ??).

Check references, some years/volumes look wired.

This work represents a novel contribution to Sb redox microbiology. If the authors can adequately address the concerns listed above with revised data and explanations, I would be pleased to recommend this work for publication.

Reviewer #2

(Remarks to the Author)
This is a novel and well-structured study reporting photoelectron-assisted microbial Sb(V) reduction (PESbR) in mining-impacted sediments. The integration of photoelectrochemical assays, controlled microcosm experiments, dual-chamber validation, metagenomic binning, metatranscriptomics, and strain-level validation is highly commendable. The figures are clear, and the concept of DOM-derived photoelectron flux in driving microbial Sb(V) reduction is significant and of broad interest to readers in environmental microbiology and bioremediation. The study provides new insights into DOM-mediated Sb(V) reduction and its environmental implications in mining-impacted sediments. Several aspects require further

clarification to improve the quality. Please find the detailed comments below.
1.Please clarify the unique role of microbes compared with purely abiotic photochemical or electrochemical Sb(V) reduction. What irreplaceable functions do microbes provide in PESbR?
2.Line 241: Author said "PESbR efficiencies were comparable to those of classical heterotrophic systems supplied with lactate (93 %) or acetate (96 %)". Please provide more direct rate comparisons between PESbR taxa and conventional heterotrophic Sb-reducers.
3.In Figure 1, humic acid showed lower performance than others. Please discuss why natural humic fractions may not always exhibit the highest photoelectrochemical activity? This may clarify the selection of humic like quinone model (AQS) as the tested DOM in this study.
4.In Figure 2, I understand the design of two microcosms, but why were two separate systems (batch vs. dual-chamber) needed? Could the dual-chamber system alone to test the essential conditions for PESbR?
5.Beyond dual-chamber results, pls provide additional evidence or discussion of how abiotic photoreduction pathways were excluded?
6.Figure 2: Consider highlighting the differences between PESbR and heterotrophic controls with clearer colors (two green bars may be confusing).
7.LN322: Provide more detail for the isolation and cultivation of these strains.
8.LN 339: It would be helpful to provide a more quantitative link between electrochemical activity and microbial metabolism. For example, try to translate the measured photocurrent values (0.10–0.18 µA cm^{-2}) into electron fluxes (e.g., mol e^{-} cm^{-2} s^{-1}).
9.The Sphingomonas and Bosea isolates showed modest reduction rates (10–30%). How do these compare to mixed-community performance? Pls clarify the differences.
10.The authors also reported analogies to arsenic (PEAsR). Could DOM-derived photoelectrons simultaneously fuel both Sb(V) and As(V) reduction, or any competitive outcomes? Since Sb–As co-contamination is common (and seen in Fig. 6b), therefore, the implications for Sb–As coexistence are suggested to be involved.
11.LN152 and 244: Provide the average completeness/contamination of MAGs, not only in the Supplementary Information.
12.Consider adding recent references on microbial uptake of extracellular electrons to better illustrate the PESbR process.
13.Consider including more datasets in supplementary data, e.g., detailed PESbR gene analysis and full field sample information.
14.Please rephrase the strong application claims. For example, instead of suggesting direct incorporation into Sb biogeochemical models and remediation strategies just using "potential implications."
15.Discussion section 1 and 2 contain repeated descriptions of DOM features.

Reviewer #3

(Remarks to the Author)
The manuscript by Zhu et al. advances photoelectron-assisted Sb(V) reduction (PESbR), positing that light-excited organics supply electrons that non-phototrophic microbes use to reduce Sb(V). Evidence spans PEC assays with natural DOM, humic-substance standards, and a quinone analog (AQS); anaerobic microcosms and a PEM dual-chamber; genome/strain data (e.g., Sphingomonas, Bosea); and a field survey linking humic-like optical signatures and photocurrents to PESbR potential. While the conceptual integration is appealing, several issues limit interpretability and generality:

Major Comments
1. Definition and units of "DOM"
"Five DOM types" include FA/HA, natural riverine/sediment DOM, and AQS; AQS is introduced as a quinone model compound rather than DOM, creating a category ambiguity. PEC doses are given as 100–400 mg L^{-1}, whereas microcosms/dual-chamber use "5 mM DOM," which is not a meaningful unit for complex mixtures and hinders comparability across experiments.
2. Sb(V) source organic-carbon confounding
Potassium antimony tartrate is used as the Sb(V) source throughout, which introduces an additional organic carbon source into the system. Given that tartrate is readily biodegradable, its presence introduces potential for confounding heterotrophic Sb(V) reduction that is not directly linked to photoelectrons or extracellular electron transfer pathways. This may limit the interpretability of observed redox activity under light/DOM conditions.
3. Dual-chamber electrochemistry and controls
The PEM-separated dual-chamber system is a central element supporting the PESbR mechanism, but the documentation of abiotic controls (e.g., sterile cathode, electrode-only chambers) is limited. The electrochemical configuration also presents inconsistencies in the reference electrode used (Ag/AgCl vs. SCE), and current–time data are not linked directly to Sb redox outcomes.
In addition, PEC Methods list a glassy-carbon working electrode with mixed geometry notation ("Ø 3 mm; 1 cm × 1 cm"), graphite counter, and Ag/AgCl reference, while a constant potential of +0.3 V vs SCE is reported; the reference scale and geometry description appear internally inconsistent.
4. Sb speciation and mass-balance accounting
Sb(III)/Sb(V) are quantified by HG-AFS (HGF-V2), yet the separation/derivatization workflow, recoveries, and DLs are not detailed. A "closed total-Sb mass balance" is asserted, and adsorption is deemed negligible at 50:1 water:sediment, but the supporting data are not presented in the main text.
5. Community/omics statistics and attibution
Community shifts are summarized (LEfSe and α/β diversity), and metatranscriptomic changes are given as fold-changes without count-based DE or FDR control; mapping/normalization details are limited, making quantitative linkage between taxa/genes and redox outcomes difficult to assess.
6. Causality for "exclusively photoelectrons"

The microcosm 4-factor matrix is clear (Light/DOM/Sb/biomass), yet the stronger causal statement that indigenous communities "rely exclusively on DOM-generated photoelectrons" precedes mechanistic isolation and remains ambitious without time-resolved light switching or EET-perturbation in the same system.

Moderate and Clarifying Comments
Illumination comparability: PEC employs a 300 W Xe lamp with AM1.5 filter, irradiance varied 400–800 W m^{-2} and distance 2–10 cm; explicit sample-plane irradiance/spectral equivalence across PEC vs microcosm/dual-chamber contexts is not shown, complicating direct comparisons.
EDTA context: PEC assays add 1 mM EDTA-2Na as a hole quencher "in all DOM assays"; the presence/absence of EDTA in biological systems is not explicitly stated, which matters given potential metal chelation/speciation effects.
pH control: Microcosm/dual-chamber sections do not report solution pH; Sb speciation and hydride-generation behavior are pH-dependent, and FA/HA dissolution can be pH sensitive.
Resazurin: Microcosms include 1‰ resazurin as an O_2 indicator; resazurin can participate in redox cycling at low concentrations, representing a potential auxiliary electron shuttle.
Field normalization: EEM region fractions and photocurrent magnitudes for field DOM are presented, but DOC normalization and sediment extraction yields are not detailed, limiting cross-site comparability.
Scope/generalization: The discussion infers broad prevalence of PESbR across sites; evidence is stronger for co-occurrence (photosensitive DOM + candidate taxa) than for quantified in situ contribution to Sb fluxes.
Figure-method alignment: The PEC section records I–t under light/dark cycling; analogous current–speciation linkage is not shown for the dual-chamber, where the causal chain is most critical.

Version 1:

Reviewer comments:

Reviewer #1

(Remarks to the Author)
The authors have addressed my comments. I have no further comments.

Reviewer #2

(Remarks to the Author)
The authors have made extensive revisions that clearly address my major concerns. The overall quality of the manuscript has been substantially improved. I therefore recommend the manuscript for acceptance, and I appreciate the authors' efforts in the revision.

Reviewer #3

(Remarks to the Author)
The reviewer appreciates the authors' revisions and the detailed rebuttal. The revised manuscript more clearly links illumination, DOM photochemistry, and microbial Sb(V) reduction. However, several issues remain that limit how confidently the main conclusions can be interpreted, especially regarding the mechanism and how broadly it applies. My key remaining points (including some new ones) are below.

1) What exactly is "DOM" in the key microcosm experiments, and how general is the claim?
A central claim is that natural DOM enables a photoelectron-driven microbial Sb(V) reduction pathway. However, the revised text and responses still leave some confusion about whether the main microcosm evidence is based on natural DOM mixtures or on a model quinone compound (AQS) used as a proxy. This affects how broadly the conclusion can be extended to natural systems and how "environmental relevance" should be read.

2) Sb(III) fate and the net environmental implication remain unclear.
The work addresses Sb(V) reduction under the experimental conditions, but the environmental meaning depends on what happens to produced Sb(III): does it stay dissolved, sorb, precipitate, or get re-oxidized? The revision discusses this qualitatively, but the study still does not strongly constrain how the observed reduction translates to Sb mobility or toxicity in intact sediments.

3) Gene/function claims: aio/ano as "Sb(III) oxidases" and bidirectional cycling may be too strong.
The revised text interprets aio/ano-type genes as Sb(III) oxidases and uses expression patterns to suggest bidirectional Sb cycling that limits Sb(III) build-up. Because aio/ano systems are best known for As(III) oxidation and substrate specificity is not guaranteed, these claims still look stronger than what the data clearly show. This affects the support for the "bidirectional Sb cycle" conclusion.

4) Resazurin and redox conditions under light cycling.
Using resazurin as a redox indicator in illuminated incubations could be seen as a confound in a study focused on redox chemistry and electron transfer. Also, light–dark cycling can shift redox conditions over time; resazurin is qualitative and

does not directly quantify O_2/Eh changes. The revision should ensure readers are convinced that redox conditions are well controlled and that the dye does not affect the outcome.

Version 2:

Reviewer comments:

Reviewer #3

(Remarks to the Author)
The MS was carefully revised. The quality was improved and meet the level of NC after 2nd revision. The reviewer would like to say "acceptable".

Open Access This Peer Review File is licensed under a Creative Commons Attribution 4.0 International License, which permits use, sharing, adaptation, distribution and reproduction in any medium or format, as long as you give appropriate credit to the original author(s) and the source, provide a link to the Creative Commons license, and indicate if changes were made.
In cases where reviewers are anonymous, credit should be given to 'Anonymous Referee' and the source.
The images or other third party material in this Peer Review File are included in the article’s Creative Commons license, unless indicated otherwise in a credit line to the material. If material is not included in the article’s Creative Commons license and your intended use is not permitted by statutory regulation or exceeds the permitted use, you will need to obtain permission directly from the copyright holder.
To view a copy of this license, visit https://creativecommons.org/licenses/by/4.0/

REVIEWER COMMENTS

Reviewer #1 (Remarks to the Author):

Microbial reduction of Sb(V) is a crucial factor in the migration of sb in mine stream sediments. This process is dependent on heterotrophic microorganisms that utilize dissolved organic matter as the carbon source. This manuscript reports a novel and environmentally significant mechanism of Sb(V) reduction mediated by non-phototrophic microorganisms utilizing photoelectrons generated from photosensitive dissolved organic matter (DOM) in sediments. This challenges the traditional understanding that Sb(V) reduction in sediments is exclusively driven by heterotrophic respiration using DOM as a carbon source. The authors adopted a series of advanced framework to support this new conceptual PESbR model. Authors demonstrate that light-excited DOM stimulates the activity of multiple non-phototrophic genera and associated redox genes, leading to enhanced Sb(V) reduction. These findings are interesting and carry significant environmental implications. The objectives of this study are clear. However, several key issues must be addressed to strengthen the clarity, environmental relevance, and broader applicability of the findings.

Response: Thanks for recognizing our work and for your valuable suggestions to improve it.

Major comments:

The use of $K_2Sb_2(C_4H_2O_6)_2$ may introduce organic tartrate, which is a readily metabolizable carbon and electron source for a wide range of heterotrophs. This complicates the interpretation that photoelectrons from DOM are the sole drivers of Sb(V) reduction. Recommend either replace with inorganic $KSb(OH)_6$ or include a tartrate-only control.

We apologize for the confusion caused by a typing error in the Methods section. In fact, all Sb(V) reduction experiments used inorganic potassium antimonate ($KSb(OH)_6$) as the Sb(V) source, rather than $K_2Sb_2(C_4H_2O_6)_2$, which we frequently use as Sb(III) stock in other studies. We have corrected the Sb(V) reagent accordingly in **line 417**. Thus, no tartrate or other organic carbon was introduced via the Sb(V) stock in the PESbR microcosms.

Supporting data for electron competition effects are lacking. Despite electron acceptor competition is briefly discussed in this manuscript, Sb is rarely the sole

terminal electron acceptor in sediments. Please provide common potential acceptor assays, this would help confirm Sb(V)'s priority.

We have addressed this point by adding additional competition assays on the basis of the PESbR microcosms. We performed parallel microcosms with common alternative terminal electron acceptors (As(V), Fe(III), NO_3^- and SO_4^{2-}). In all cases, the presence of these competing acceptors led to a decrease in Sb(V) reduction, as reflected by lower Sb(III) accumulation (**Figure S6**).

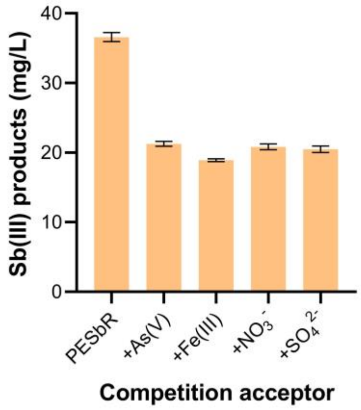

Figure S6. The addition of alternative terminal electron acceptors suppresses photoelectron-driven Sb(V) reduction, as reflected by the decreased Sb(III) products.

These results also support that Sb(V) competes with other terminal electron acceptors in sediments, consistent with our original evidence for respiratory diversification in PESbR members (**Fig. 4a** and **Fig. 5b**). Accordingly, we have added the statement in **line 206**:

> *"In line with this, the addition of other terminal electron acceptors suppressed photoelectron-driven Sb(V) reduction (Figure S6), which supports the presence of photoelectron-acceptor competition."*

Although three rivers were tested for PESbR occurrence, but all are close to Xikuangshan. Readers will wonder if PESbR is specific to this area. Could the authors include additional representative Sb-contaminated rivers or conduct a wider comparison to increase the environmental relevance? This would increase the ecological scope.

We appreciate this insightful comment on the broader environmental relevance of PESbR.

Initially, we have quickly screened several Sb-contaminated sites from other representative

mining area across different provinces and/or ore belts. In multiple sites, short-term illumination (~9 days) indeed stimulated microbial Sb(V) reduction relative to dark controls, indicating that PESbR-like responses are not confined to Xikuangshan and can also occur in other Sb-impacted sediments. However, because the Xikuangshan region represents one of the most typical and extensive Sb mining-impacted watersheds with persistently elevated Sb loading, we thus focused the main-text microcosm dataset on Xikuangshan for consistency and representativeness.

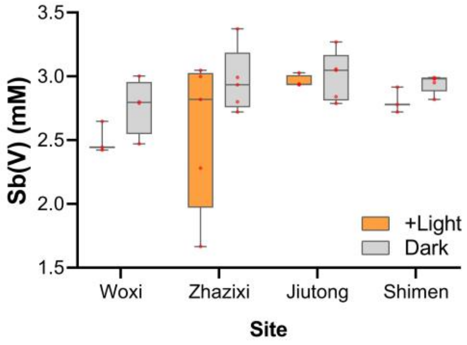

Figure R1. Multi-site microcosm assays highlighting the wide occurrence of PESbR potential across distinct Sb-contaminated sediments.

In the revision, to further assess the broader biogeographic distribution of PESbR potential, we performed a comparative genomic analysis using public NCBI reference genomes for the key PESbR-associated genera *Bosea* and *Sphingomonas*. In total, 14 *Bosea* and 161 *Sphingomonas* genomes from diverse geographic locations were analyzed (**Figure S9**). Comparative analysis of our MAGs and these reference genomes revealed similarly abundant Sb(V)-reducing genes (*anrA*, *arsC* and *arrA*), suggesting that the genetic potential for Sb(V) reduction via the proposed PESbR pathway is widespread.

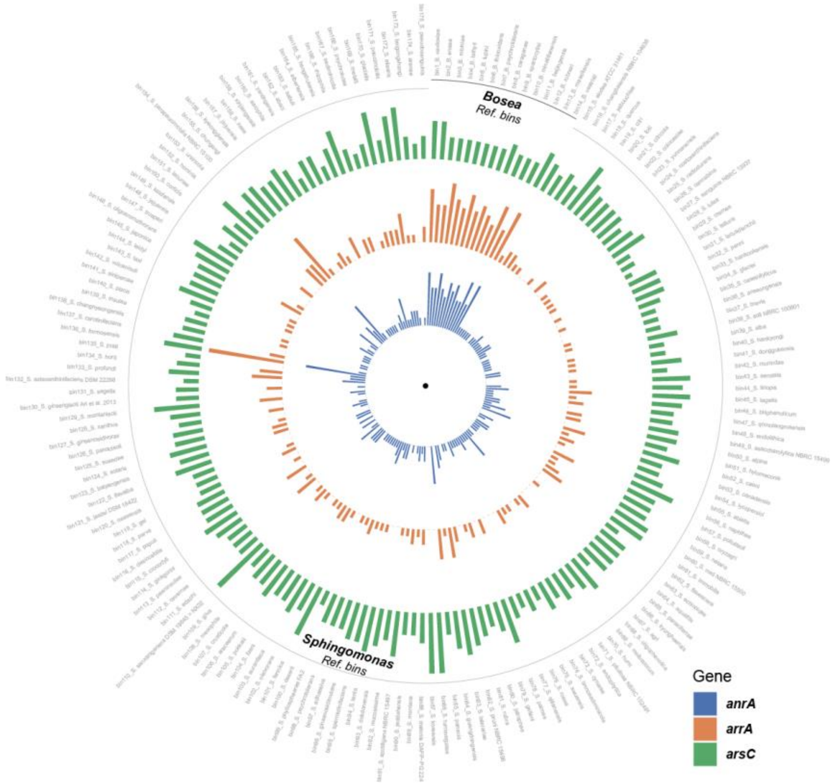

Figure S9. Comparative genomic analysis of NCBI reference genomes of PESbR-associated taxa (*Bosea* and *Sphingomonas*) showing metabolic potentials comparable to PESbR-associated MAGs, as evidenced by abundant key Sb(V)-reducing genes.

This cross-validation have been incorporated as supplementary data and discussion in :

> ***<u>Line 228</u>:** "Furthermore, a comparative genomic analysis of public NCBI reference genomes of Bosea and Sphingomonas also revealed similarly abundant anrA, arsC and arrA genes, suggesting that PESbR potential are likely widespread within these genera."*

The manuscript should better discuss the key environmental variables that modulate PESbR, such as pH, seasonal DOM pulses, sediment optical depth, light penetration (turbidity, shading), algal blooms, and suspended particles. Identifying the conditions in which PESbR is favored or constrained would help indicate its real-world impact and inform models or remediation strategies.

Done. We added a brief Discussion (**<u>line 280</u>**) to clarify the environmental controls on PESbR.

> "Notably, natural variability in DOM concentration and photoreactivity [1], pH, light penetration depth [2], suspended particles [3], and hydrodynamics may affect PESbR activity. Future work will need to evaluate these environmental factors and their implications for

Sb(V) mobility.”

DOM is reported in mg L^{-1} in the electrochemical section and in mM in microcosm incubations, which is confusing. Please standardize it across all sections. This will allow comparison with environmental conditions.

We appreciate this suggestion. In the electrochemical assays, some samples were extracted from complex environmental mixtures (e.g., sediment DOM) with no defined molar mass, so it can only be reported as mg L^{-1}. In contrast, the microcosms used standards/model compounds with known molecular weights, for which mM is appropriate and concise. We have now standardized units as much as possible across the manuscript.

For the microcosm light intensity, how was the light intensity chosen? Does it reflect natural irradiance levels in sediment–water interfaces? Please justify the representativeness of light settings. Also, whether the high-intensity near-source illumination (e.g., 800 W m^{-2} at 2 cm) could have introduced thermal artifacts or oxygen intrusion?

We thank the reviewer for these important details.

The light intensity of 800 W m^{-2} was chosen to approximate natural solar irradiance and corresponded to a daily radiant exposure of ~23 MJ m^{-2} d^{-1}, which falls within the reported annual range of surface solar radiation (6–26 MJ m^{-2} d^{-1}) for the study region [4].

Potential thermal artifacts were minimized by conducting all incubations in a temperature-controlled room (25 ± 1 °C) with intermittent illumination and air conditioning.

To prevent oxygen intrusion, microcosm bottles were sealed with butyl rubber stoppers, the headspace was flushed with N_2, and resazurin was included as a redox indicator and monitored throughout the experiments; it remained colorless, indicating that oxygen intrusion was negligible under our setup.

Accordingly, we have added the clarification to the Methods section (**line 418**):

> *“Bottles were flushed with N_2 (100 mL min^{-1} for 30 min), sealed with butyl rubber stoppers and supplemented with 1 ‰ resazurin as an oxygen indicator. The indicator remained colorless throughout the incubations, indicating minimal oxygen intrusion. All incubations were incubated at 25 ± 1°C under 12 h light/12 h dark cycles at 800 W m^{-2},*

following illumination setting used for photocurrent assays."

PESbR is described as distinct from semiconductor-driven or phototrophic reduction. Try to clarify this to prior known studies particularly in terms of microbial phototrophy or electroactive processes.

Done. We now clarify how PESbR's feature differs from microbial phototrophy and semiconductor-driven hybrid systems.

Canonical phototrophy relies on intracellular photosystems and pigments in phototrophic organisms that capture light and generate reducing power within the cell. While semiconductor-driven biohybrids instead use solid-phase minerals or engineered semiconductors (e.g., Fe oxides, TiO_2, electrodes) as light absorbers that generate excited electrons that are transferred to attached microorganisms. By contrast, PESbR, as defined here, involves non-phototrophic sediment bacteria that capture extracellular photoelectrons released from naturally occurring photosensitive DOM. PESbR do not require complex photosystems or solid semiconductors, which may represent a more broadly distributed and naturally occurring metabolic mode.

We have added a statement in **line 266** to clarify this distinction:

"Besides, PESbR appears distinct from canonical phototrophy and engineered semiconductor-based biohybrids, which rely on intracellular photosystems in phototrophs or on solid minerals and engineered semiconductors as light absorbers. Instead, non-phototrophic PESbR communities use photosensitive DOM as a dissolved light absorber that releases extracellular photoelectrons to drive Sb(V) reduction. This pattern may represent a more broadly distributed and naturally occurring metabolic mode in sediments."

Current isolate experiments show low reduction (~10–30%), but no dark controls are provided. Proving these strains exhibit PESbR needs stronger evidence. Additionally, the statement that reduction rates are comparable to heterotrophic respiration is misleading—heterotrophic controls reportedly reach >90%. In addition, try to include cell growth or Sb tolerance for these strains.

We appreciate these three suggestions.

We initially did not include dark controls for the isolates because batch microcosm experiments (**Figure 2a**) had already shown that Sb(V) reduction does not occur in the

absence of light. Following the reviewer's suggestion, we have now added dark controls for the *Bosea* and *Sphingomonas* strains in the revision. Under dark conditions, their Sb(V) reduction was only trace (<1%), in contrast to the 10–30% reduction observed under illumination. These data are now presented in the revised **Figure 5d** and provide stronger evidence that these strains can perform PESbR.

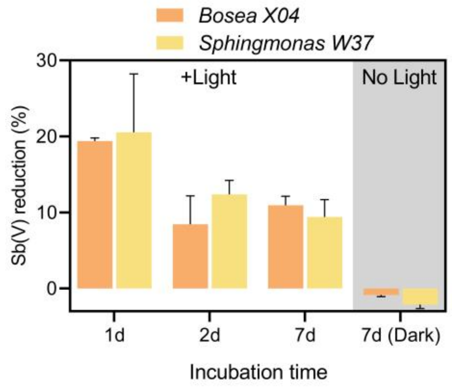

Figure 5d. Sb(V) reduction by the isolates in pure-culture microcosms containing photosensitive DOM under light–dark cycles versus dark controls.

Second, we agree that our previous statement comparing reduction rates was overstated. We have removed this wording and now describe the isolates as exhibiting measurable PESbR capacity relative to classical heterotrophic rates.

Third, following the reviewer's suggestion, we have added Sb tolerance and growth data for the *Bosea* and *Sphingomonas* strains (**Figure S7**), which support their proposed role as PESbR-capable taxa.

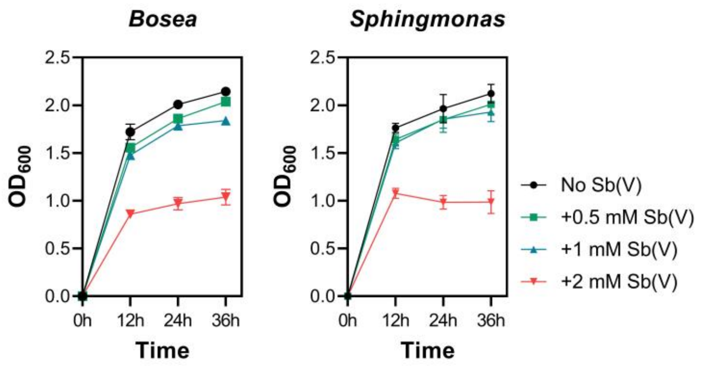

Figure S7. Growth of *Bosea* and *Sphingomonas* strains under different Sb(V) stress.

The difference of reduction rates between isolates and communities is intriguing.

The superior performance of mixed communities over isolates suggests the presence of microbial cooperation. It is suggested to consider possible co-occurrence or interaction involving Pseudomonas and Sphingomonas and other “helper strains” in detoxification, or EET facilitation or cofactors.

We thank the reviewer for this insightful suggestion. Following the reviewer’s suggestion, we analyzed co-occurrence patterns between the main PESbR taxa (orange nodes) and other community members (pink nodes). The network shows numerous positive links at the community level, suggesting multiple interactions that may support Sb(V) reduction. Several closely associated taxa (e.g., *Terrimonas*, *Flavobacterium*, *Desulfovibrio*, *Pseudomonas*, *Burkholderia, Anaeromyxobacter*, *Comamonas*) may act as putative “helper” strains by contributing to community cooperation, metal detoxification, extracellular electron transfer, and redox-active cofactor production. Such cooperative functions could help explain why mixed communities exhibit higher Sb(V) reduction rates than isolates.

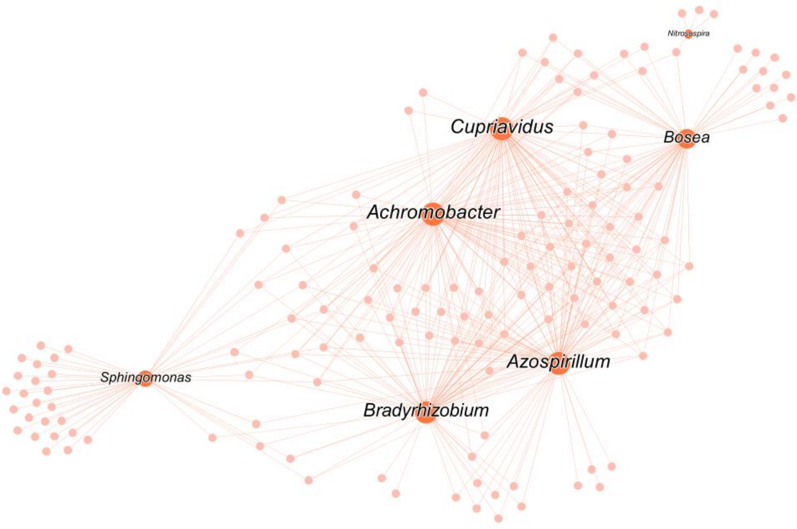

Figure S12. Co-occurrence network showing numerous positive links between main PESbR taxa (orange nodes) and other community members (pink nodes).

Accordingly, we have added this analysis and a brief discussion in **line 322**:

> *“Co-occurrence network analysis further revealed numerous positive associations between the major PESbR taxa and other community members (Figure S12), suggesting potential cooperation during Sb(V) reduction. Such interaction likely contributes to the higher Sb(V) reduction observed in mixed communities (Figure 2b) compared with individual isolates (Figure 5d).”*

The comparison of anrA and arrA genes involved in PESbR pathway is important. Discuss the functional differences between two pathways in Sb(V) reduction and whether they are associated with DOM.

PESbR involves two molybdopterin oxidoreductases, *anrA* and *arrA*. In our MAGs, *anrA* is enriched in core PESbR populations (**Figure 4b**) and shows stronger transcriptional responses (**Figure 5b**), which indicates a dedicated Sb(V) respiratory reductase that is closely coupled to DOM-derived photoelectrons. In contrast, *arrA* is more broadly distributed and responds weakly to light and DOM, suggesting a more general metalloid respiration pathway that provides background Sb(V)/As(V) reduction in the community. We have now added a short paragraph in **line 192** to clarify the respective roles of *anrA* and *arrA* in the PESbR pathway.

> *"Core Sb(V) reductase genes and regulators responded strongly, including anrA, arsC, arrR/arsR increased by up to ~2.8 fold. While arrA expression remained similar to controls, possibly reflecting its lower specificity for Sb(V) in an Sb-only system."*

The reported photoelectron current from field DOM are valuable. How to correlate such electron generation with estimated Sb(V) reduction potential?

We attempted a simple estimate to link the measured photoelectron current to the potential Sb(V) reduction in field samples. The measurable photocurrent densities of field DOM (~0.10–0.18 μA cm^{-2}, **Figure 6d**) correspond to an electron flux on the order of ~10^{-4}–10^{-3} mol e^{-} m^{-2} d^{-1}. This flux could in principle support Sb(V) reduction rates roughly of a 10–100 mg Sb(V) m^{-2} d^{-1} at the illuminated water interface. This order-of-magnitude estimate indicates that DOM-derived photoelectron fluxes are quantitatively capable of sustaining the PESbR rates observed in our experiments.

But we have clarified that natural systems are highly complex. Variations in DOM composition and other factors will likely lower the actual in situ Sb(V) reduction rates relative to this theoretical value. Our current field dataset is therefore insufficient to establish a robust quantitative relationship between photocurrent and Sb flux. Accordingly, we only added a brief statement in **line 239** to involve this calculation.

> *"All DOM extracts produced stable photocurrents (~0.10–0.18 μA cm^{-2}) under illumination, indicating that photosensitive DOM is common in these mine-impacted sediments. These photocurrents flux could in principle support Sb(V) reduction of roughly 10–100 mg Sb(V) m^{-2} d^{-1} at the illuminated water interface."*

The manuscript does not clearly distinguish whether Sb(III) is present in dissolved, sorbed, or mineralized forms. Suggest adding data to determine the speciation and environmental fate of reduced Sb. This will help evaluate the remediation potential of PESbR.

We thank the reviewer for this suggestion. In this study, because the microcosms used a relatively high water-to-sediment ratio (50:1, **line 429**), we primarily quantified aqueous Sb(V)/Sb(III). A near-closed total-Sb mass balance (**Figure S2**) indicates that significant sorption and mineralization losses were unlikely under these conditions, and thus Sb(III) was present mainly in dissolved form in our experiments.

Besides, the environmental fate of reduced Sb has been extensively studied [5]. Sb(III) produced from Sb(V) reduction can persist as dissolved $Sb(OH)_3$, be sorbed or co-precipitated with Fe/Mn (oxyhydr)oxides and organic matter, or form Sb(III)-bearing minerals such as stibnite (Sb_2S_3) under sulfidic conditions. Therefore, the net remediation potential of PESbR ultimately depends on how these pathways are balanced in real sediments. The distribution of Sb(III) between dissolved, sorbed and mineral phases remains to be resolved in future work.

PESbR is mainly proposed to occur in sediments, but how deep can light penetrate? Sediments may do not receive much incident sunlight. Could this process be occurred in overlying waters? Try to consider the depth limitations in natural systems.

We thank the reviewer for raising this concern regarding the penetrate depth. Initially, we have clarified that samples were collected from shallow surface sediments (0–10 cm below the waterline; **line 398**). This design was chosen for exactly the consideration raised by the reviewer: the deeper and darker sediment layers are unlikely to receive sufficient light to support efficient PESbR. Thus, our conclusions refer primarily to light-exposed surface sediments rather than to deeper dark horizons.

In addition, we agree that PESbR could in principle also occur in the overlying water, which contains suspended particles, photosensitive DOM, Sb(V), and some PESbR-associated taxa while light is still available. However, the overlying water is strongly diluted and experiences frequent hydraulic disturbances, which likely weaken the establishment of stable PESbR consortia compared with the more concentrated and physically stable sediments. This is consistent with our observations that PESbR-associated genera are less abundant in overlying waters than in paired sediments (**Figure 6e–f**).

Therefore, our results suggest that PESbR is most effective in shallow, light-exposed surface

sediments, which act as a hotspot. While a smaller contribution from the overlying water cannot be excluded. We now highlight this depth limitation in the revised manuscript:

> **Line 246:** *"These genera were more enriched in sediments (mean total ~2.36%) than in the overlying water column (~0.18%, Figure 6f), indicating habitat-specific niches and suggesting light-exposed surface sediments as likely hotspots for PESbR activity."*

> **Line 280**: *"Notably, natural variability in DOM concentration and photoreactivity, pH, light penetration depth, suspended particles, and hydrodynamics may affect PESbR activity. Future work will need to evaluate these environmental factors and their implications for Sb(V) mobility."*

Figure. 3 clearly shows the distinct members responsible for different microcosms, but why not show taxa enriched in no DOM (light-only) controls? These could represent members prefer to light. Including this would help identify phototroph-enriched but PESbR-independent taxa.

These taxa have indeed been analyzed and are provided in the Supplementary Information (**Figure S4**). Because the main focus of **Figure 3** is to highlight the PESbR-favorable communities, those phototroph-enriched but PESbR-independent taxa are only presented as a reference and used for comparison with the PESbR-enriched taxa shown in **Figure 3**. Therefore, no change has been made here.

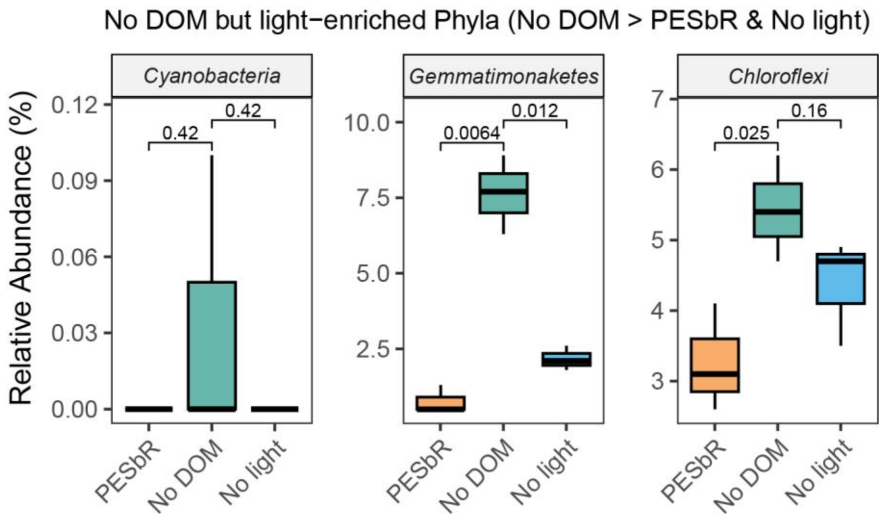

Figure S4. Relative abundances of phototrophic taxa uniquely enriched in the light only, DOM absent control microcosms.

Fig. 6: Try to provide DOM classification with more informative analysis beyond the simple 3D-EEM clusters.

Done. Following the approach [6], we complemented the EEM results with fluorescence index and ratio analyses to further characterize the sources and compositional properties of sediment DOM. These results are now provided in **Figure S11**.

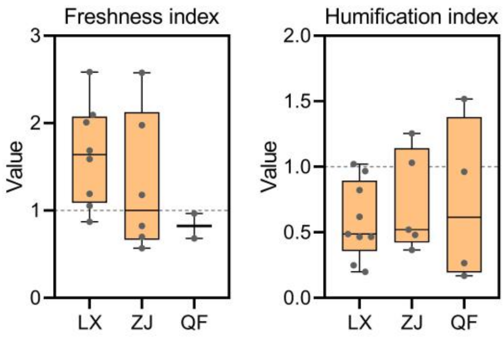

Figure S11. Fluorescence index–based characterization of DOM putative sources and compositional properties, using the freshness index (FI) and humification index (HIX).

It would help the reader to include a schematic overview of the full experimental workflow (currently missing in Figure S1).

Done. We have added a new schematic diagram summarizing the full workflow to guide readers through the multi-step PESbR experimental framework, from DOM/electrochemical characterization to batch microcosms, omics analyses, isolate-level validation, and field cross-validation (**Figure S1**).

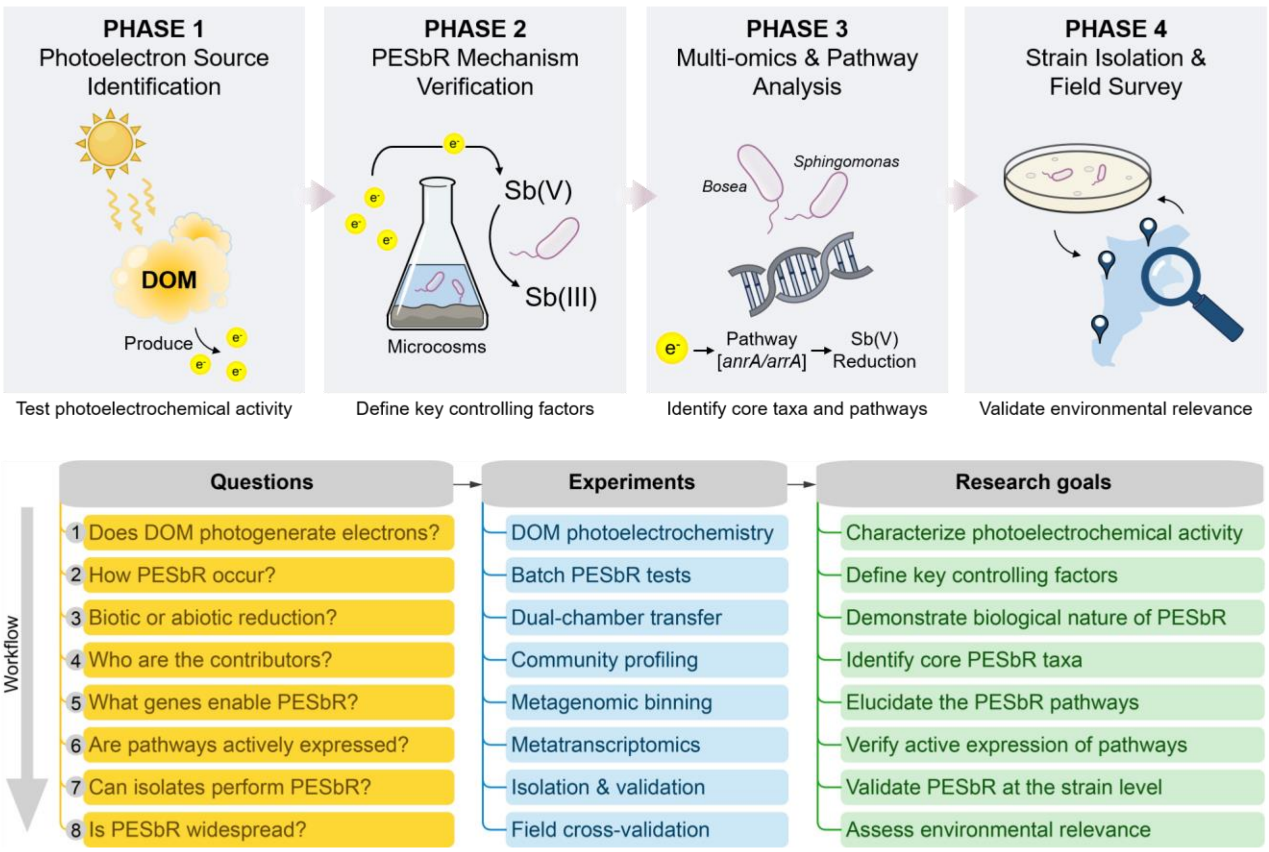

Figure S1. Study workflow demonstrating that DOM-generated photoelectrons enable microbial Sb(V) reduction in mine-stream sediments. Steps include DOM photoelectrochemical assays, batch and dual-chamber microcosms, multi-omics profiling, isolation and testing of PESbR strains, and field-scale cross-validation.

How was light calibration performed?

The light intensity was measured and calibrated using a light density meter (CEL-NP2000−2, Ceaulight, Beijing, China) [7]. In addition, throughout the experiments, the distance and position of the light source relative to the reactors were fixed to ensure uniform and stable illumination. We have added this description in **line 411**:

> *"Illumination was calibrated with a light density meter and adjusted …"*

How were Rayleigh scatters removed in EEM data?

We now clarify that Rayleigh scatter bands were removed from all EEMs using a Delaunay triangulation interpolation within a ±20 nm excision window around the first- and second-

order Rayleigh scatter lines. This procedure was implemented using the ‘DOMFluor’ and ‘drEEM’ toolbox in the software of MATLAB2022.

Why was EDTA-2Na chosen as the quencher, and what are its potential effects on DOM behavior?

EDTA-2Na was selected as the quencher because it is water-soluble has abundant lone pairs of electrons, which tend to react with electron-deficient holes, and widely used as a hole scavenger [8, 9]. It can quickly consume photogenerated oxidative holes and thereby enhance the measured photocurrent, which allows us to confirm that the observed signals indeed originate from the photoexcitation of DOM.

Notably, we clarified that EDTA-2Na was only added in the DOM photoelectrochemical assays (**Figure 1**) and was not used in any subsequent batch PESbR microcosms or biological incubations (**Figure 2**). Therefore, it does not influence DOM behavior in the biological experiments reported here.

Need more discussion and comparison of Pseudomonas and Sphingomonas’ role in Sb reduction. Current statement is rough.

We appreciate this comment. Throughout the manuscript, we focus on two key PESbR taxa, *Sphingomonas* and *Bosea*. We therefore suspect that the mentioned “*Pseudomonas*” may be a typing error here? As this genus does not appear in the main text.

Regarding *Sphingomonas* and *Bosea*, we agree that their roles in Sb reduction are important. However, to our knowledge, previous studies have mainly linked these genera to Sb(III) oxidation rather than Sb(V) reduction. Thus, there is very limited literature for a detailed comparison of their Sb-reducing roles.

Meanwhile, our multi-line evidence collectively suggests that *Sphingomonas* and *Bosea* are major drivers in PESbR system, as supported by (i) they are significantly enriched in PESbR microcosms (**Figure 3c,d**); (ii) their MAGs harbor abundant PESbR core genes (**Figure 4a**); (iii) multiple *Sphingomonas* and *Bosea* isolates from in situ samples exhibit clear PESbR activity (**Figure 5c–e**); and (iv) their enrichment in field sediments from Sb-contaminated sites (**Figure 6e**).

Following the reviewer’s suggestion, we have expanded their Discussion (**line 319**) to better emphasize this point:

"For example, previous studies have mainly linked Sphingomonas and Bosea to Sb(III) oxidation, Sb resistance and plant-associated functions in Sb-contaminated habitats, whereas their roles in Sb(V) respiratory reduction or other dissimilatory metal(loid) reduction pathways have rarely been documented. Co-occurrence network analysis further revealed numerous positive associations between the major PESbR taxa and other community members (Figure S12), suggesting potential cooperation during Sb(V) reduction. Such interaction likely contributes to the higher Sb(V) reduction observed in mixed communities (Figure 2b) compared with individual isolates (Figure 5d)."

Please clarify how PESbR strains were "efficiently" isolated from sediment slurry.

Done. We clarified that PESbR strains were isolated using a modified high-throughput cultivation strategy [10, 11], which greatly increased the probability of obtaining single PESbR strains from the sediment slurry. Briefly, 2 g of fresh sediment was homogenized in sterile medium to prepare a well-dispersed suspension. A limiting dilution (on the order of ~10^6-fold) was then prepared and dispensed into approximately 30 sterile 96-well plates, assuming that cells in each well follow a Poisson distribution. Under these conditions, most inoculated wells are expected to contain either 0 or 1 viable cell, providing a high likelihood of single-strain growth in each turbid well.

After incubation, those wells showing visible turbidity were randomly selected, and the isolates were subjected to a PESbR activity assay under the same conditions as the batch microcosms. Wells that exhibited PESbR activity were then subjected to taxonomy identification, and confirmed pure candidate strains were retained for further analysis. Using this workflow, we approximately obtained on the order of >1,000 isolates within about one month, thereby efficiently obtaining the desirable PESbR strains for this study.

We have supplemented the details in **line 459**:

"A modified high-throughput cultivation protocol was used to effectively screen Sb-tolerant microbes from sediment slurries. Sediment slurries were subjected to ~10^6-fold limiting dilution into multiple 96-well plates supplemented with 0.1 mM Sb stress to obtain single-cell inocula, followed by screening of turbid wells for PESbR activity. Candidate isolates were identified by 16S rRNA gene sequencing. Six isolates belonging to two key PESbR genera (Sphingomonas and Bosea) were obtained."

It is suggested to provide quantitative rate data (μmol Sb L^{-1} h^{-1}) in addition to % efficiency.

Done. We have calculated the quantitative Sb(V) reduction rates as suggested in **line 107**:

> *"… reduction efficiencies reached 53%–71% at initial Sb(V) concentrations of 0.2–1 mM, corresponding to average volumetric rates of ~24~72 μmol Sb L^{-1} d^{-1}."*

The manuscript would benefit from a clearer discussion of the practical implications of PESbR.

Our primary goal in this study is to describe a previously unrecognized pathway of PESbR. Thus mainly emphasized: (i) the potential importance of DOM-derived photoelectron fluxes in shaping element redox cycling in mine-impacted environments; and (ii) the need to better understand how Sb(V) reduction proceeds in natural sediments, where such processes are likely active but remain poorly understood.

Indeed, as the reviewer notes, PESbR may represent a low-carbon, naturally occurring metabolic pathway with promising practical implications. We therefore briefly note that by interventions targeting DOM quality and light penetration, could be used to enhance PESbR activity and thereby promote in situ Sb(III) attenuation. Such strategy may provide a potential conceptual basis for low-carbon wastewater or mining-water treatment.

However, we also acknowledge that our current data are not sufficient to support such detailed application design. Therefore, we only added a short Discussion (**line 384**) to clarify the potential practical relevance of PESbR.

> *"… under controlled illumination together with co-precipitation approaches (e.g., sulfide or iron-oxides) are helpful to retain reduced Sb(III) in situ, which may provide a conceptual basis for low-carbon wastewater or mining-water treatment."*

In the dual-chamber systems, the reduction rate of Sb(V) at the biocathode was relatively low compared to batch microcosms. Why? Which factors may hinder the efficiency?

This is mainly attributed to the different system configurations. In the dual-chamber setup, the anode and cathode are separated by a proton exchange membrane (**line 433**). This setup greatly limits both electron and proton transfer from DOM to the microbes, finally leading to slower Sb(V) reduction than in a well-mixed batch microcosm.

Unify terminology throughout the maintext.

Done.

Fix unit formatting ($\mu A\ cm^{-2}$, $W\ m^{-2}$, $mg\ C\ L^{-1}$).

Done.

Correct coordinates formatting (27°78′ N ??).

Done.

Check references, some years/volumes look wired.

Done.

This work represents a novel contribution to Sb redox microbiology. If the authors can adequately address the concerns listed above with revised data and explanations, I would be pleased to recommend this work for publication.

We sincerely appreciate your positive evaluation and insightful comments, which have greatly strengthened the quality of this manuscript.

Reviewer #2 (Remarks to the Author):

This is a novel and well-structured study reporting photoelectron-assisted microbial Sb(V) reduction (PESbR) in mining-impacted sediments. The integration of photoelectrochemical assays, controlled microcosm experiments, dual-chamber validation, metagenomic binning, metatranscriptomics, and strain-level validation is highly commendable. The figures are clear, and the concept of DOM-derived photoelectron flux in driving microbial Sb(V) reduction is significant and of broad interest to readers in environmental microbiology and bioremediation. The study provides new insights into DOM-mediated Sb(V) reduction and its environmental implications in mining-impacted sediments. Several aspects require further clarification to improve the quality. Please find the detailed comments below.

Response: We sincerely thank the reviewer for the positive evaluation and constructive suggestions. Following your comments, we have carefully revised the manuscript. The point-by-point responses are provided below.

1.Please clarify the unique role of microbes compared with purely abiotic photochemical or electrochemical Sb(V) reduction. What irreplaceable functions do microbes provide in PESbR?

Done. We now more clearly compare PESbR with purely abiotic photochemical or electrochemical Sb(V) reduction and clarify the unique role of microbes.

Abiotic photochemical and electrochemical systems can reduce Sb(V), but they typically rely on engineered materials (e.g., electrodes, semiconductors, added reductants) and external energy input and maintenance, which limits their applicability in natural sediments. In contrast, PESbR uses naturally occurring, photosensitive DOM as the light absorber and native sediment microbes as the catalyst, making it more compatible with natural environments and low-input field conditions. Microbes further provide specific electron-transfer pathways and respiratory oxidoreductases (e.g., Anr, Arr) for selective Sb(V) reduction, rather than nonspecific abiotic reduction. In terms of in situ applicability, PESbR also allows Sb(V) attenuation without additional chemical amendments or the risk of secondary pollution. Overall, we suggest that PESbR complements, rather than replaces, abiotic photochemical and electrochemical technologies and primarily functions as a low-carbon, self-sustaining pathway in natural systems.

We have added a brief statement regarding these features in **line 75** :

"This pattern makes PESbR a low-input, self-sustaining pathway for Sb(V) reduction that may complement purely abiotic photochemical and engineered electrochemical pathway in natural systems."

2.Line 241: Author said "PESbR efficiencies were comparable to those of classical heterotrophic systems supplied with lactate (93 %) or acetate (96 %)". Please provide more direct rate comparisons between PESbR taxa and conventional heterotrophic Sb-reducers.

Done. According to previous studies, classical heterotrophic Sb(V)-respiring bacteria (e.g., *Sinorhizobium* sp. JUK-1, *Desulfuribacillus stibiiarsenatis* MLFW-2, *Dechloromonas* sp. AR-2 and *Propionivibrio* sp. AR-3) can achieve Sb(V) reduction rates on the order of ~20–30 μmol Sb(V) L^{-1} h^{-1} under ideal conditions with abundant electron donors, nutrients, pure cultures and strictly anoxic environments. For comparison, in our PESbR microcosms where 53–71% of 0.2–1 mM Sb(V) was reduced over 7 days, corresponding to average rates of approximately 1–3 μmol Sb(V) L^{-1} h^{-1}. We have now revised the relevant statement in **line 107** to clarify this rate comparison.

"Over 7 days, reduction efficiencies reached 53%–71% at initial Sb(V) concentrations of 0.2–1 mM, corresponding to average volumetric rates of ~24~72 μmol Sb L^{-1} d^{-1} … … microcosms amended with lactate/acetate showed 93–96% Sb(V) reduction (Figure 2b), which is in line with reported rates for classical heterotrophic Sb(V)-reducers."

3.In Figure 1, humic acid showed lower performance than others. Please discuss why natural humic fractions may not always exhibit the highest photoelectrochemical activity? This may clarify the selection of humic like quinone model (AQS) as the tested DOM in this study.

The lower photoelectrochemical response (**Figure 1**) of humic acid is mainly attributed to its optical properties rather than an inherently weak chromophore composition. The commercial humic acid solution used here is highly colored and strongly light-absorbing/scattering, which makes the suspension showed a noisier and less smooth photocurrent signal than other DOM types.

Given that DOM is a chemically heterogeneous mixture, we thus chose AQS as a humic-like quinone model rather than bulk humic acid for the microcosm experiments (**Figure 2**) to more cleanly identify the role of photoelectrons. AQS was selected because (i) it is a well-established analogue of humic-like chromophores with strong photoreactivity [12, 13], and (ii)

it remains sufficiently transparent in solution to allow more homogeneous illumination.

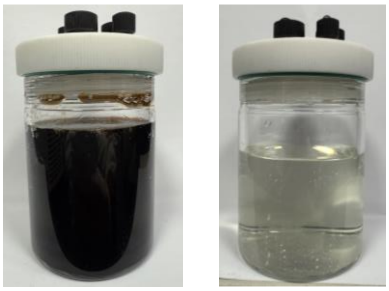

Humic acid AQS

Figure R2. Comparison of light transmittance between the commercial humic acid and AQS solutions used in this study.

Together, we have briefly clarified this point in **line 258:**

> *"…several DOM types exhibited measurable photoelectrochemical activity, and that photocurrent depended on molecular composition, concentration and light intensity."*

4.In Figure 2, I understand the design of two microcosms, but why were two separate systems (batch vs. dual-chamber) needed? Could the dual-chamber system alone to test the essential conditions for PESbR?

These two setups were designed to serve complementary purposes rather than to duplicate each other. In principle, the dual-chamber system alone indeed can test the essential conditions for PESbR, as it involves generation, transfer, and capture of photoelectron for microbial Sb(V) reduction. However, the introduction of a proton exchange membrane inevitably increases mass-transfer limitations, thus leads to a relatively low observed Sb(V) reduction (**Figure 2d**). Therefore, we also employed single-chamber batch microcosms with controlled factors, in which DOM, microbes and Sb(V) remain in direct contact, allowing more efficient interactions and clearer Sb(V) reduction responses.

5.Beyond dual-chamber results, pls provide additional evidence or discussion of how abiotic photoreduction pathways were excluded?

We agree that excluding abiotic photoreduction pathways is critical for defining PESbR. Thus, in the single-chamber microcosms, we specifically included autoclaved controls with DOM and light but no active biomass. In this group, Sb(V) reduction remained <1% (**Figure 2b**),

indicating that abiotic photoreduction contributes negligibly. In addition, the strong enrichment of specific PESbR taxa and the upregulation of respiratory reductases further support the biological nature of PESbR rather than an abiotic pathway. We have clarified this point in **line 261**:

> *"These rates indicate that DOM-derived photoelectrons alone can make a measurable contribution to Sb(V) bioreduction under experimental conditions … confirming the biological nature of PESbR rather than an abiotic photochemical process."*

6.Figure 2: Consider highlighting the differences between PESbR and heterotrophic controls with clearer colors (two green bars may be confusing).

Done.

7.LN322: Provide more detail for the isolation and cultivation of these strains.

Done. We clarified that PESbR strains were isolated using a modified high-throughput cultivation strategy [10, 11], which greatly increased the probability of obtaining single PESbR strains from the sediment slurry. Briefly, 2 g of fresh sediment was homogenized in sterile medium to prepare a well-dispersed suspension. A limiting dilution (on the order of ~10^6-fold) was then prepared and dispensed into approximately 30 sterile 96-well plates, assuming that cells in each well follow a Poisson distribution. Under these conditions, most inoculated wells are expected to contain either 0 or 1 viable cell, providing a high likelihood of single-strain growth in each turbid well.

After incubation, those wells showing visible turbidity were randomly selected, and the isolates were subjected to a PESbR activity assay under the same conditions as the batch microcosms. Wells that exhibited PESbR activity were then subjected to taxonomy identification, and confirmed pure candidate strains were retained for further analysis. Using this workflow, we approximately obtained on the order of >1,000 isolates within about one month, thereby efficiently obtaining the desirable PESbR strains for this study.

We have supplemented the details in **line 459**:

> *"A modified high-throughput cultivation protocol was used to effectively screen Sb-tolerant microbes from sediment slurries. Sediment slurries were subjected to ~10^6-fold limiting dilution into multiple 96-well plates supplemented with 0.1 mM Sb stress to obtain single-cell inocula, followed by screening of turbid wells for PESbR activity.*

Candidate isolates were identified by 16S rRNA gene sequencing. Six isolates belonging to two key PESbR genera (Sphingomonas and Bosea) were obtained."

8.LN 339: It would be helpful to provide a more quantitative link between electrochemical activity and microbial metabolism. For example, try to translate the measured photocurrent values (0.10–0.18 μA cm^{-2}) into electron fluxes (e.g., mol e^{-} cm^{-2} s^{-1}).

Thanks for this helpful suggestion. We now convert the photocurrent into electron fluxes. The photocurrent densities of field DOM (~0.10–0.18 μA cm^{-2}, **Figure 6d**) correspond to an electron flux on the order of ~10^{-4}–10^{-3} mol e^{-} m^{-2} d^{-1}. We added this calculation in **line 239**:

"All DOM extracts produced stable photocurrents (~0.10–0.18 μA cm^{-2}) under illumination, indicating that photosensitive DOM is common in these mine-impacted sediments (Figure 6d). These photocurrents flux could in principle support Sb(V) reduction of roughly 10–100 mg Sb(V) m^{-2} d^{-1} at the illuminated water interface."

9.The Sphingomonas and Bosea isolates showed modest reduction rates (10–30%). How do these compare to mixed-community performance? Pls clarify the differences.

The community-level PESbR performance (53–71%) exceeds that of individual strains of *Sphingomonas* and *Bosea* (10–30%). This is reasonable because *Sphingomonas* (~9%) and *Bosea* (~4%) together account for only a fraction of the putative PESbR community and coexist with many other taxa involved in substrate transformation and redox processes. These genera are likely tightly associated with multiple community members, suggesting that microbial interactions may enhance overall Sb(V) reduction.

10.The authors also reported analogies to arsenic (PEAsR). Could DOM-derived photoelectrons simultaneously fuel both Sb(V) and As(V) reduction, or any competitive outcomes? Since Sb–As co-contamination is common (and seen in Fig. 6b), therefore, the implications for Sb–As coexistence are suggested to be involved.

We agree that photoelectrons can fuel both Sb(V) and As(V) bioreduction. Sb and As are

chemically analogous, and previous studies have shown that As(V) reductases (e.g., Arr, Ars) can also reduce Sb(V). Consistent with this, our PESbR MAGs carry arrA and arsC, in addition to anrA, suggesting a shared or overlapping Sb–As redox pathway.

Following the reviewer's suggestion, we have added supplementary data showing that the presence of As(V) can indeed influence Sb(V) reduction, as reflected by decreased Sb(III) accumulation. This pattern suggests the potential competition for photoelectrons and/or shared reductases.

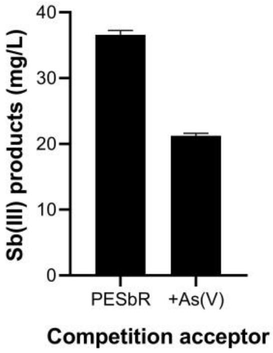

Figure S6. Co-existing As(V) as the electron acceptor suppress photoelectron-driven Sb(V) reduction.

We also acknowledge that, in natural Sb–As co-contaminated sediments, competition or co-reduction is likely and will depend on relative Sb/As concentrations and enzyme abundances. Although our data support the possibility that photoelectrons can simultaneously fuel Sb(V) and As(V) reduction, our microcosm design was primarily optimized for Sb(V) and did not allow us to resolve As(V)/As(III) speciation in detail. The exact allocation of photoelectron flux between Sb and As therefore remains beyond the scope of this study and would be addressed in future work.

We have now added a brief paragraph in **line 334** to clarify the implications of coexistence:

> *"In natural Sb–As co-contaminated sediments, DOM-derived photoelectrons are likely shared between Sb(V) and As(V) reduction, which reflect both the ubiquity of metal-dependent photoelectrophy and the distinct biogeochemical behaviors of these two analogues."*

11.LN152 and 244: Provide the average completeness/contamination of MAGs, not only in the Supplementary Information.

Done. We have now reported the MAGs quality in **line 144**.

12.Consider adding recent references on microbial uptake of extracellular electrons to better illustrate the PESbR process.

Done. Attached the ref 43#.

13.Consider including more datasets in supplementary data, e.g., detailed PESbR gene analysis and full field sample information.

Done. Following the suggestion, we revised the presentation of Tables S2–S4 and expanded the number of supplementary figures from 7 to 13.

14.Please rephrase the strong application claims. For example, instead of suggesting direct incorporation into Sb biogeochemical models and remediation strategies just using “potential implications.”

Done. We have carefully rephrased all overly strong statements throughout the manuscript.

15.Discussion section 1 and 2 contain repeated descriptions of DOM features.

We have removed or merged redundant descriptions in these two sections.

Reviewer #3 (Remarks to the Author):

The manuscript by Zhu et al. advances photoelectron-assisted Sb(V) reduction (PESbR), positing that light-excited organics supply electrons that non-phototrophic microbes use to reduce Sb(V). Evidence spans PEC assays with natural DOM, humic-substance standards, and a quinone analog (AQS); anaerobic microcosms and a PEM dual-chamber; genome/strain data (e.g., Sphingomonas, Bosea); and a field survey linking humic-like optical signatures and photocurrents to PESbR potential. While the conceptual integration is appealing, several issues limit interpretability and generality:

Response: We thank the reviewer for this overall assessment and constructive suggestions. We have revised the manuscript to improve both the interpretability and generality in this work.

Major Comments

1. Definition and units of “DOM”

“Five DOM types” include FA/HA, natural riverine/sediment DOM, and AQS; AQS is introduced as a quinone model compound rather than DOM, creating a category ambiguity. PEC doses are given as 100–400 mg L^{-1}, whereas microcosms/dual-chamber use “5 mM DOM,” which is not a meaningful unit for complex mixtures and hinders comparability across experiments.

For the category issue, we initially examined five representative types (including FA, HA, natural riverine/sediment DOM, and AQS) to illustrate that diverse DOM types exhibit photoelectrochemical activity (**Figure 1**). However, given that DOM is composed of thousands of individual organic molecules with high complexity and heterogeneity [14], we specifically chose AQS as a humic-like quinone model rather than bulk humic acid for the microcosm experiments (**Figure 2**) to more cleanly identify the role of photoelectrons. AQS is a well-established analogue of humic-like analogue with strong photoreactivity and is therefore widely used in similar studies [12, 13]. This rationale is now clarified in **line 404**:

> *“… the humic-like quinone model compound anthraquinone-2-sulfonate (AQS; 99.99 %, Aladdin Co.,) …”*

For the unit issue, in the electrochemical assays, some samples were extracted from complex environmental mixtures (e.g., sediment DOM) with no defined molar mass, so it can only be reported as mg L^{-1}. In contrast, the microcosms used standards/model compounds with

known molecular weights, for which mM is appropriate and concise. We have now standardized units as much as possible across the manuscript.

2. Sb(V) source organic-carbon confounding

Potassium antimony tartrate is used as the Sb(V) source throughout, which introduces an additional organic carbon source into the system. Given that tartrate is readily biodegradable, its presence introduces potential for confounding heterotrophic Sb(V) reduction that is not directly linked to photoelectrons or extracellular electron transfer pathways. This may limit the interpretability of observed redox activity under light/DOM conditions.

We apologize for the confusion caused by a typing error in the Methods section. In fact, all Sb(V) reduction experiments used inorganic potassium antimonate ($KSb(OH)_6$) as the Sb(V) source, rather than $K_2Sb_2(C_4H_2O_6)_2$, which we frequently use as Sb(III) stock in other studies. We have corrected the Sb(V) reagent accordingly in **line 417**. Thus, no tartrate or other organic carbon was introduced via the Sb(V) stock in the PESbR microcosms.

3. Dual-chamber electrochemistry and controls

The PEM-separated dual-chamber system is a central element supporting the PESbR mechanism, but the documentation of abiotic controls (e.g., sterile cathode, electrode-only chambers) is limited. The electrochemical configuration also presents inconsistencies in the reference electrode used (Ag/AgCl vs. SCE), and current–time data are not linked directly to Sb redox outcomes.

We thank the reviewer for these three important comments.

First, we used the single-chamber batch microcosms as the primary framework to establish the essential conditions for PESbR. The corresponding controls (no DOM, no light, no Sb(V), or no live biomass) showed <1% Sb(V) reduction (**Figure 2a–b**), indicating that abiotic pathways are negligible, which has indicated that abiotic pathways are negligible. Thus, the dual-chamber cell was employed in a complementary setup to demonstrate the chain of photoelectron generation, transfer, and microbial capture (**Figure 2c–d**), by decoupling direct contact between DOM and microbes present in the single-chamber microcosms. In addition, given the strong mass-transfer limitations imposed by the proton exchange membrane, any abiotic Sb(V) reduction in the dual-chamber configuration would be expected to be even smaller than the single-chamber abiotic controls. Taken together, an additional

set of abiotic controls in the dual-chamber cell was therefore not necessary.

Second, there are no inconsistencies in the reference electrodes used in the dual-chamber electrochemistry and PEC assays, because the purposes and operating modes of the two configurations are fundamentally different. The single-chamber PEC assays were designed to characterize DOM photoactivity under well-defined potential control, and therefore employed a three-electrode setup with an Ag/AgCl reference electrode. In contrast, the dual-chamber system was operated as a two-electrode cell without any externally applied potential, and thus did not require or include a reference electrode.

Third, we agree that more direct links between current–time data and Sb redox outcomes would be helpful. However, due to the experimental configuration, continuous PEC measurements and Sb(V)-reducing microcosms could not be performed in the same dual-chamber setup. Instead, photocurrent responses and Sb(V) reduction were analyzed in parallel and complementary experiments, which together demonstrate the PESbR process. Therefore, in the revision, we added supplementary data from a time-resolved light–dark switching experiment (24 h per cycle) conducted in the same Sb(V)-reducing microcosms (**Figure S8**). Sb(V) reduction rates dropped markedly during dark periods and recovered upon light, which could link light-dependent current dynamics to Sb redox outcomes.

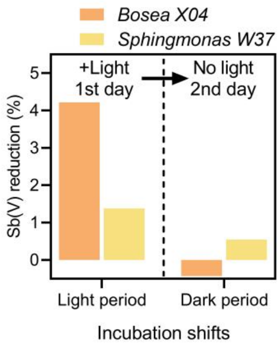

Figure S8. Sb(V) reduction dynamics under time-resolved light–dark switching.

In addition, PEC Methods list a glassy-carbon working electrode with mixed geometry notation ("Ø 3 mm; 1 cm × 1 cm"), graphite counter, and Ag/AgCl reference, while a constant potential of +0.3 V vs SCE is reported; the reference scale and geometry description appear internally inconsistent.

We have corrected this inconsistency in **line 409**:

> *"A constant potential of +0.3 V versus Ag/AgCl was applied."*

4. Sb speciation and mass-balance accounting

Sb(III)/Sb(V) are quantified by HG-AFS (HGF-V2), yet the separation/derivatization workflow, recoveries, and DLs are not detailed. A "closed total-Sb mass balance" is asserted, and adsorption is deemed negligible at 50:1 water:sediment, but the supporting data are not presented in the main text.

First, we now added the quantification details in **line 426**:

> *"… for Sb speciation (Sb(III), Sb(V), total Sb) by hydride-generation atomic fluorescence spectrophotometer (HG-AFS) with a recovery rate >90% and detection limit (> 0.01 μg/L). Ar gas (≥ 99.999%) was used as the carrier gas (300 mL/min) and shield gas (800 mL/min)."*

Second, the supporting data for the total-Sb mass balance is shown in **Figure S2-b**, which support the adsorption is deemed negligible in the microcosms. This has stated in **line 109**:

> *"… total-Sb remained nearly constant over time, suggesting limited sorption losses in microcosms (Figure S2)."*

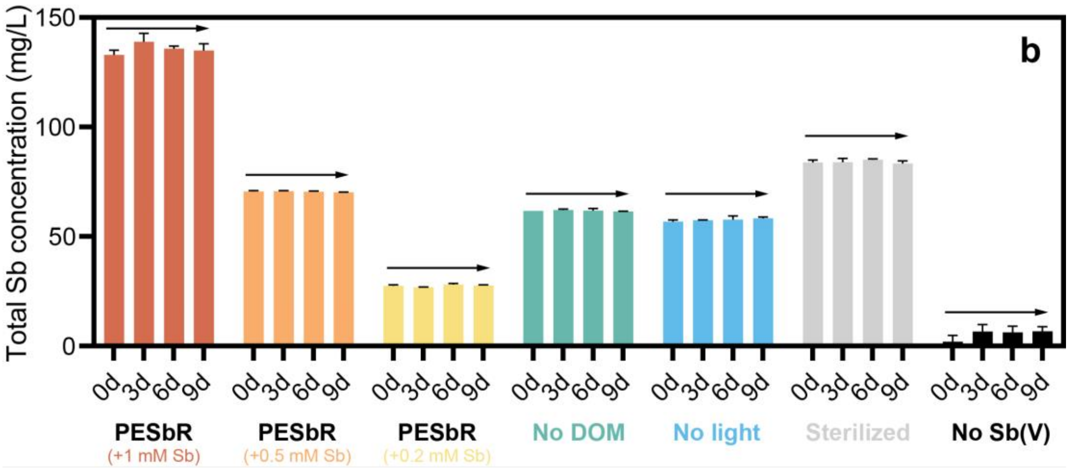

Figure S2. (b) Total Sb concentration remains constant in all microcosms over time, demonstrating mass balance and excluding significant adsorption or release from the sediment solid phase.

5. Community/omics statistics and attibution

Community shifts are summarized (LEfSe and α/β diversity), and metatranscriptomic changes are given as fold-changes without count-based DE or FDR control; mapping/normalization details are limited, making quantitative linkage between taxa/genes and redox outcomes difficult to assess.

We appreciate this important comment.

First, we have added more method-appropriate statistical details as suggested.

Second, we clarify that putative PESbR taxa were identified from 16S rRNA amplicon data rather than from the metatranscriptomes, so the concern about reads' mapping/normalization does not apply here. Metatranscriptomic data were only used to characterize functional pathway responses. In addition, our main purpose was to identify PESbR-associated taxa and pathways rather than to construct a fully quantitative predictive model. Accordingly, PESbR-associated taxa and redox outcomes were linked through a complementary combination of amplicon-based community shifts, MAGs recovery, and strain-level validation, while quantitative attribution of Sb(V) reduction to individual populations or genes is acknowledged to be beyond the scope of this study.

6. Causality for "exclusively photoelectrons"

The microcosm 4-factor matrix is clear (Light/DOM/Sb/biomass), yet the stronger causal statement that indigenous communities "rely exclusively on DOM-generated photoelectrons" precedes mechanistic isolation and remains ambitious without time-resolved light switching or EET-perturbation in the same system.

We thank the reviewer for this important suggestion. We have added supplementary data from a time-resolved light–dark switching experiment (24 h per cycle) conducted in the same Sb(V)-reducing microcosms (**Figure S8**). It is shown that Sb(V) reduction rate indeed dropped markedly when switch to dark cycle, which together support the conclusion that 4-factors is necessary for the PESbR activity. This result has been incorporated into **line 222**. We have also revised the original strong phrasing throughout the text as suggested.

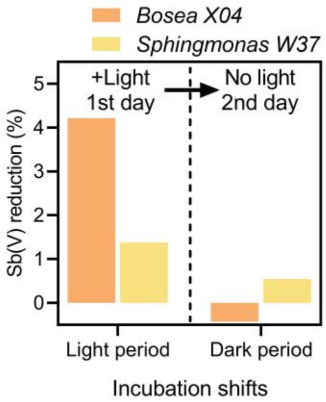

Figure S8. Sb(V) reduction dynamics under time-resolved light–dark switching.

Moderate and Clarifying Comments

Illumination comparability: PEC employs a 300 W Xe lamp with AM1.5 filter, irradiance varied 400–800 W m^{-2} and distance 2–10 cm; explicit sample-plane irradiance/spectral equivalence across PEC vs microcosm/dual-chamber contexts is not shown, complicating direct comparisons.

These illumination conditions were kept consistent across the PEC assays, batch microcosms and dual-chamber systems. We added a clarifying statement in **line 420**:

> *"All incubations were incubated at 25 ± 1°C under 12 h light/12 h dark cycles at 800 W m^{-2}, following illumination setting used for photocurrent assays."*

EDTA context: PEC assays add 1 mM EDTA-2Na as a hole quencher "in all DOM assays"; the presence/absence of EDTA in biological systems is not explicitly stated, which matters given potential metal chelation/speciation effects.

We appreciate this important point. We clarified that EDTA-2Na was added only in the DOM photoelectrochemical assays (**Figure 1**) and was not used in any subsequent PESbR microcosms or biological incubations (**Figure 2**). Therefore, EDTA does not influence metal speciation or DOM behavior in the biological experiments.

pH control: Microcosm/dual-chamber sections do not report solution pH; Sb

speciation and hydride-generation behavior are pH-dependent, and FA/HA dissolution can be pH sensitive.

We agree that pH strongly affects Sb speciation and DOM behavior. In this study, all experiments were conducted under near-neutral conditions to minimize interferences. We have added this detail to the Methods section (**line 417**).

Resazurin: Microcosms include 1‰ resazurin as an O_2 indicator; resazurin can participate in redox cycling at low concentrations, representing a potential auxiliary electron shuttle.

We thank the reviewer for raising this point. In fact, resazurin was added at the same concentration to all microcosms, so any potential effect would be identical across treatments and not bias the comparison among light/DOM/biomass conditions. In addition, the no-DOM controls showed <1% Sb(V) reduction (**Figure 2b**), indicating that resazurin alone did not support measurable Sb(V) reduction under our conditions. Moreover, the 1‰ resazurin dosage corresponds to a commonly used low concentration, which its contribution to electron transfer can be negligible.

Field normalization: EEM region fractions and photocurrent magnitudes for field DOM are presented, but DOC normalization and sediment extraction yields are not detailed, limiting cross-site comparability.

Done. We have added these yields in **line 236** to provide a basis for cross-site comparability. Specifically, sediment DOM extraction yields ranged from 20–530 mg DOC kg^{-1} (dry weight), corresponding to approximately 6–160 mg DOC kg^{-1} (wet weight) assuming a 70% moisture content.

Scope/generalization: The discussion infers broad prevalence of PESbR across sites; evidence is stronger for co-occurrence (photosensitive DOM + candidate taxa) than for quantified in situ contribution to Sb fluxes.

We appreciate this comment. We have therefore toned down statements about the prevalence and dominance of PESbR as suggested.

Figure-method alignment: The PEC section records I–t under light/dark cycling; analogous current–speciation linkage is not shown for the dual-chamber, where the causal chain is most critical.

We agree that similar records in the dual-chamber system would be informative. However, due to the dual-chamber configuration, continuous PEC measurements could not be performed in that setup. Instead, photocurrent responses were characterized in PEC assays, while the dual-chamber system were analyzed in parallel and complementary experiments to support the same causal chain. These two setups together demonstrate the PESbR process. In the revision, we added supplementary data from a time-resolved light–dark switching experiment (24 h per cycle) conducted in the same Sb(V)-reducing microcosms (**Figure S8**). Sb(V) reduction rates dropped markedly during dark periods and recovered upon light, which could link light-dependent current dynamics to Sb redox outcomes.

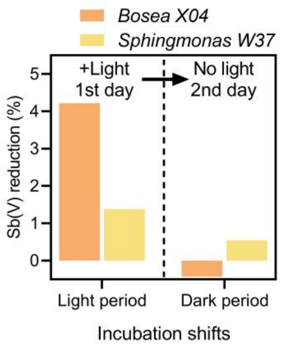

Figure S8. Sb(V) reduction dynamics under time-resolved light–dark switching.

References:

1. Berg SM*, et al.* Dissolved organic matter photoreactivity is determined by its optical properties, redox activity, and molecular composition. *Environmental Science & Technology* **57**, 6703-6711 (2023).
2. Huang S*, et al.* Sunlight Significantly Enhances Soil Denitrification via an Interfacial Biophotoelectrochemical Pathway. *Environmental Science & Technology* **57**, 7733-7742 (2023).
3. Kang W*, et al.* Marine Colloids Boost Nitrogen Fixation in Trichodesmium erythraeum by Photoelectrophy. *Environmental Science & Technology* **58**, 9236-9249 (2024).
4. Zeng Z*, et al.* Daily global solar radiation in China estimated from high-density meteorological observations: A random forest model framework. *Earth and Space Science* **7**, e2019EA001058 (2020).
5. Zhang Y*, et al.* Reaction pathways and Sb(III) minerals formation during the reduction of Sb(V) by Rhodoferax ferrireducens strain YZ-1. *Journal of hazardous materials* **465**, 133240 (2024).
6. Zhang B*, et al.* Transformation of dissolved organic matter during full-scale treatment of integrated chemical wastewater: Molecular composition correlated with spectral indexes and acute toxicity. *Water Research* **157**, 472-482 (2019).
7. Huang S*, et al.* Dissolved organic matter acting as a microbial photosensitizer drives photoelectrotrophic denitrification. *Environmental Science & Technology* **56**, 4632-4641 (2022).
8. Guo Z*, et al.* Novel photoelectron-assisted microbial reduction of arsenate driven by photosensitive dissolved organic matter in mine stream sediments. *Environmental Science & Technology* **58**, 22170-22182 (2024).
9. Ding H*, et al.* Tailoring photocatalytic performance through EDTA-2Na coordination: Competition of electron transfer to conduction band and valence band in bismuth-based compounds. *Applied Surface Science* **695**, 162866 (2025).
10. Gao H*, et al.* Chemolithoautotrophic antimonite oxidation coupled nitrogen fixation in the rhizosphere of local plant in antimony tailing area. *Environmental Science & Technology* **59**, 12703-12716 (2025).
11. Zhang J*, et al.* High-throughput cultivation and identification of bacteria from the plant root microbiota. *Nature Protocols* **16**, 988-1012 (2021).
12. Hong YG*, et al.* Humic substances act as electron acceptor and redox mediator for microbial dissimilatory azoreduction by Shewanella decolorationis S12. *Journal of microbiology and biotechnology* **17**, 428-437 (2007).
13. Chen M*, et al.* Anthraquinone-2-Sulfonate as a microbial photosensitizer and capacitor drives solar-to-N2O production with a quantum efficiency of almost unity. *Environmental Science & Technology* **56**, 5161-5169 (2022).
14. Cui Y*, et al.* Unveiling the dark matter of riverine dissolved organic matter and its role in molecular chemodiversity. *Water Research* **289**, 124870 (2026).

REVIEWER COMMENTS

Reviewer #1 (Remarks to the Author):
The authors have addressed my comments. I have no further comments.

Response: We thank Reviewer #1 for the careful review and for confirming that our revisions have addressed all concerns.

Reviewer #2 (Remarks to the Author):
The authors have made extensive revisions that clearly address my major concerns. The overall quality of the manuscript has been substantially improved. I therefore recommend the manuscript for acceptance, and I appreciate the authors' efforts in the revision.

Response: We thank Reviewer #2 for the careful review and for confirming that our revisions have addressed all concerns.

Reviewer #3 (Remarks to the Author):
The reviewer appreciates the authors' revisions and the detailed rebuttal. The revised manuscript more clearly links illumination, DOM photochemistry, and microbial Sb(V) reduction. However, several issues remain that limit how confidently the main conclusions can be interpreted, especially regarding the mechanism and how broadly it applies. My key remaining points (including some new ones) are below.

Response: We thank Reviewer #3 for the constructive evaluation and for recognizing the improvements. We respond to each remaining point below and revised the manuscript accordingly.

1) What exactly is "DOM" in the key microcosm experiments, and how general is the claim?
A central claim is that natural DOM enables a photoelectron-driven microbial Sb(V) reduction pathway. However, the revised text and responses still leave some confusion about whether the main microcosm evidence is based on natural DOM mixtures or on a model quinone compound (AQS) used as a proxy. This affects how broadly the conclusion can be extended to natural systems and how "environmental relevance" should be read.

Response: We appreciate the reviewer's request to more clearly distinguish natural DOM mixtures from the model compound used in specific assays. We agree that it is both important and challenging to balance mechanistic control with the inherent heterogeneity [1] of DOM. We therefore revised the manuscript to state explicitly what was used in each section and to clarify that the strength of PESbR is expected to depend on DOM composition and

geochemistry.

We have now clarified that the microcosm (Fig. 2) used AQS as a representative photosensitizer because (i) it usually serves as a model quinone redox mediator and proxy for quinone-like redox-active moieties in natural DOM [2, 3]; (ii) it provides a controlled mechanistic proxy to test whether photoelectron supply can support microbial Sb(V) reduction, while minimizing uncertainty introduced by bulk DOM variability; (iii) it is not intended as a growth substrate, thereby reducing the carbon-source confounding; and (iv) it remains sufficiently transparent in solution to allow more homogeneous illumination.

Revised Method **Line 406:**

> *"Single chamber anaerobic PESbR microcosms (100 mL serum bottles) contained … 5 mM photosensitizer (AQS used here) and… AQS was used as a defined quinone redox mediator (proxy for DOM quinone-like moieties in natural DOM) to isolate photoelectron transfer while minimizing DOM heterogeneity and carbon-substrate confounding."*

Revised Results **Line 103:**

> *"Batch microcosms were constructed to include … photosensitizer (AQS, used here as a quinone proxy for redox-active moieties in DOM), and … (Figure 2a)."*

To further support environmental relevance beyond the AQS proxy, we strengthened and clarified the following evidences based on natural DOM and field-relevant samples: (i) diverse natural DOM isolates show measurable photoelectrochemical activity under illumination (Fig. 1a), indicating that DOM-derived photoelectrons can be available in environmental settings; (ii) across multiple Sb-impacted sites, microcosms using native sediments without AQS addition (i.e., relying on indigenous DOM) show reproducible light-stimulated Sb(V) reduction relative to dark controls (Fig. R1), supporting that PESbR-like response is not restricted to the laboratory system; (iii) contaminated sediments show a consistent set of conditions that are expected to favor PESbR, including elevated Sb, humic-like DOM signatures, photocurrent responses from sediment-extracted DOM, and the putative PESbR-associated genera (Fig. 6); and (iv) comparative genomics of *Bosea* and *Sphingomonas* from diverse locations supports that key Sb(V)-reduction genes are common within these PESbR-genera (Fig. S9), while we note that gene presence alone does not demonstrate in situ activity.

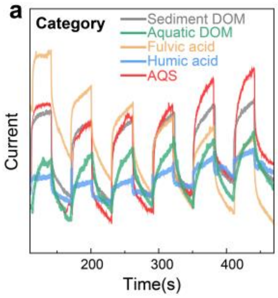

Figure 1a. Photoelectrochemical activity of DOM under light–dark cycling.

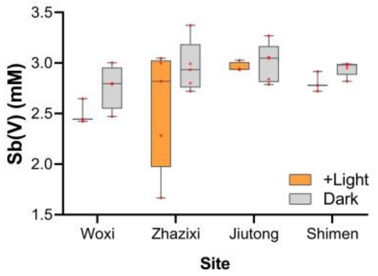

Figure R1. Multi-site microcosm assays across Sb-impacted sediments. Native sediments without AQS addition (relying on indigenous DOM) show reproducible light-stimulated Sb(V) reduction relative to dark controls.

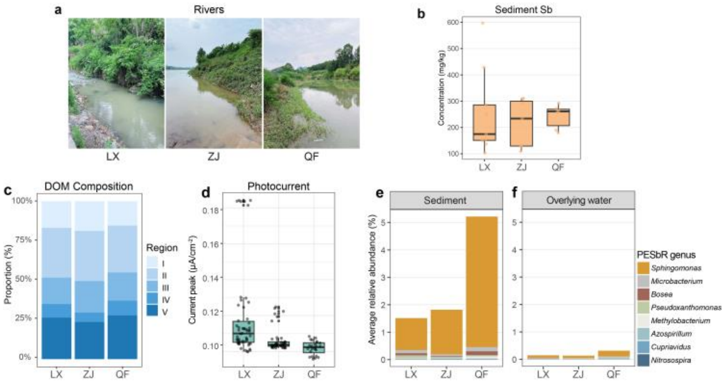

Figure 6. Field-scale cross-validation of PESbR potential in contaminated sediments.

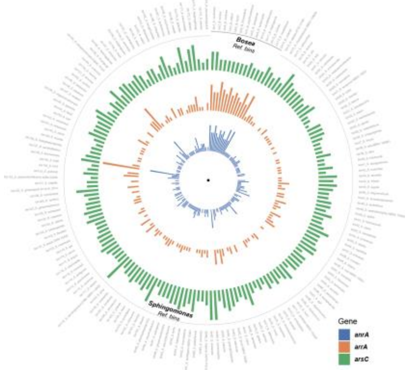

Figure S9. Comparative genomic analysis of public NCBI reference genomes from two PESbR-enriched genera (*Bosea* and *Sphingomonas*) shows gene repertoires broadly consistent with the recovered MAGs (e.g., *anrA*/*arsC*/*arrA*), suggesting that genetic potential for Sb redox transformations may be common within these genera.

2) Sb(III) fate and the net environmental implication remain unclear.

The work addresses Sb(V) reduction under the experimental conditions, but the environmental meaning depends on what happens to produced Sb(III): does it stay dissolved, sorb, precipitate, or get re-oxidized? The revision discusses this qualitatively, but the study still does not strongly constrain how the observed reduction translates to Sb mobility or toxicity in intact sediments.

Response: We agree that Sb(III) fate is critical for interpreting the net environmental implications of Sb(V) reduction. In our microcosms, Sb speciation shifts (Fig. 2) and dissolved total Sb (Fig. S2) were measured in 0.22 μm–filtered supernatants, and these dissolved fractions remained near-constant under the high water-to-sediment ratio (50:1). This indicates that substantial net transfer of Sb from the dissolved pool to sorbed or mineralized forms was not evident over the incubation period.

In addition, we also assessed Sb(III) re-oxidation using a short-term oxic stability assay with end-point samples containing reduced Sb(III) (Fig. R2). Over 24 h, it showed measurable Sb(III)→Sb(V) re-oxidation (~30 μmol L^{-1} d^{-1}) under oxic exposure, whereas sterilized oxic controls showed negligible changes.

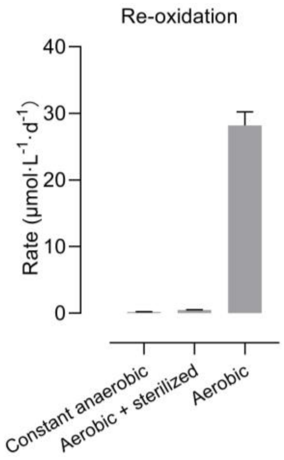

Figure R2. Oxic exposure triggers measurable microbial Sb(III)→Sb(V) re-oxidation in PESbR end-point supernatants (sterile vs. non-sterile controls).

We also agree that in intact sediments the net impact on Sb mobility and toxicity will depend on site-specific conditions (e.g., Fe/Mn (oxyhydr)oxides, sulfides, and organic matter) and redox dynamics [4]. However, this study we mainly focus on the photoelectron-driven speciation shift thus discuss Sb(III) fate only qualitatively. We therefore revised the manuscript to avoid over-interpreting mobility/toxicity from the microcosms and emphasize that PESbR primarily demonstrates a light-enabled Sb speciation shift, while the net effect on Sb mobility/toxicity is context dependent in natural settings.

Revised Discussion **Line 374:**

> *"... should quantify solid-phase Sb speciation and Sb(III) re-oxidation under redox fluctuations to assess the net implications of PESbR for Sb mobility and toxicity."*

3) Gene/function claims: aio/ano as "Sb(III) oxidases" and bidirectional cycling may be too strong.

The revised text interprets aio/ano-type genes as Sb(III) oxidases and uses expression patterns to suggest bidirectional Sb cycling that limits Sb(III) build-up. Because aio/ano

systems are best known for As(III) oxidation and substrate specificity is not guaranteed, these claims still look stronger than what the data clearly show. This affects the support for the "bidirectional Sb cycle" conclusion.

Response: We thank the reviewer for this important clarification and agree that our previous wording was stronger than warranted. The *aio* system is best characterized for As(III) oxidation; despite it has been suggested as a proxy to infer potential Sb(III)-oxidation capacity [5], substrate specificity and functional contribution can vary across taxa and conditions. We therefore revised the manuscript to refer it as "aio-type arsenite oxidase-like gene (potentially involved in Sb oxidation)" rather than as definitive Sb(III) oxidases.

For *anoA*, prior work has linked *anoA*-like genes to Sb(III) oxidation in specific strains [6]; however, public database annotations remain limited and functional equivalence across taxa cannot be assumed. We therefore describe our dataset as a "putative *anoA*-like Sb(III)-oxidation gene" and have toned down "bidirectional Sb cycling" to "potential redox flexibility" throughout the manuscript.

Revised Results **Line 159:**

> *"Redox homeostasis and potential redox flexibility: … Notably, MAGs also encoded aio-type arsenite oxidase-like genes (aioA/aioB) and putative anoA-like genes, which is consistent with potential redox versatility that may limit intracellular Sb(III) excessive accumulation."*

Revised Discussion **Line 350:**

> *"One possible interpretation is that these oxidation-associated markers reflect broader redox versatility in PESbR process."*

4) Resazurin and redox conditions under light cycling.

Using resazurin as a redox indicator in illuminated incubations could be seen as a confound in a study focused on redox chemistry and electron transfer. Also, light–dark cycling can shift redox conditions over time; resazurin is qualitative and does not directly quantify O_2/Eh changes. The revision should ensure readers are convinced that redox conditions are well controlled and that the dye does not affect the outcome.

Response: Resazurin was used only as an anoxia indicator and was added at the same low level to all microcosms (1‰, ~4 μM), so it should not bias comparisons among light/DOM/biomass treatments.

To further test whether the dye affects Sb(V) reduction, we ran parallel PESbR microcosms with and without resazurin and observed no statistically significant difference for Sb(V) reduction rates (Fig. R3; Welch t-test, $p = 0.10$). In addition, resazurin did not lead to Sb(V)

reduction, where sterilized controls and no-AQS treatments showed no measurable Sb(V) reduction despite the presence of resazurin.

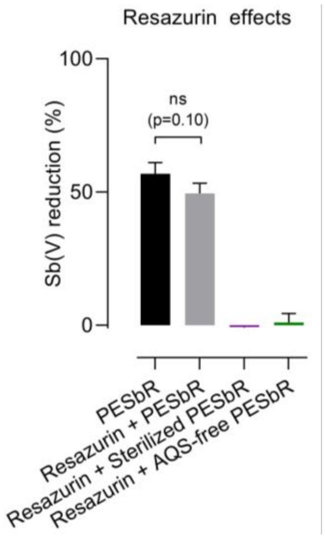

Figure R3. Effect of resazurin on Sb(V) reduction in PESbR microcosms.

Redox conditions also maintained stable throughout the incubations. Microcosms were pre-incubated to consume residual oxidants and reach a stable anoxic state. After this pre-equilibration, ORP remained around −90 ± 10 mV, with minor shift during subsequent light/dark cycling (Fig. R4). Together, these results indicate that resazurin did not confound the outcome and that redox conditions were stable during incubation.

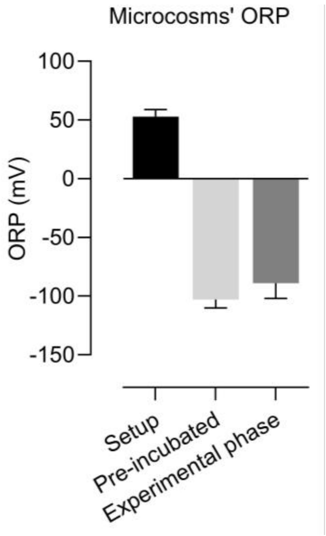

Figure R4. ORP in microcosms at setup, after pre-incubation, and during the experimental light/dark cycling phase.

Revised Method **Line 411:**

> *"... and supplemented with resazurin (~4 μM) as an anoxia indicator. The indicator remained colorless throughout the incubations, indicating minimal oxygen intrusion. ORP remained stable with minor change during incubations."*

References

1. Cui Y, et al. Unveiling the dark matter of riverine dissolved organic matter and its role in molecular chemodiversity. Water Research 289, 124870 (2026).
2. Hong YG, et al. Humic substances act as electron acceptor and redox mediator for microbial dissimilatory azoreduction by shewanella decolorationis s12. Journal of Microbiology and Biotechnology 17, 428-437 (2007).
3. Chen M, et al. Anthraquinone-2-sulfonate as a microbial photosensitizer and capacitor drives solar-to-n2o production with a quantum efficiency of almost unity. Environmental Science & Technology 56, 5161-5169 (2022).
4. Zhang Y, et al. Reaction pathways and sb(iii) minerals formation during the reduction of sb(v) by rhodoferax ferrireducens strain yz-1. Journal of Hazardous Materials 465, 133240 (2024).
5. Min D, Liu D-F. It is time to explore unique mechanisms for microbial antimony conversion rather than relying on analogies with arsenic bioconversion. Environmental Science & Technology 57, 15780-15781 (2023).
6. Li J, et al. Proteomics and genetics for identification of a bacterial antimonite oxidase in agrobacterium tumefaciens. Environmental Science & Technology 49, 5980-5989 (2015).

REVIEWER COMMENTS

Reviewer #3 (Remarks to the Author):

The MS was carefully revised. The quality was improved and meet the level of NC after 2nd revision. The reviewer would like to say "acceptable".

Response: We thank Reviewer #3 for the positive assessment and for confirming that our second revision has addressed all remaining concerns.
